# Comparative catalytic insights into green and commercial gold nanoparticles: synergistic catalytic reduction of organic pollutants

**DOI:** 10.1039/d5ra09335j

**Published:** 2026-02-26

**Authors:** Md. Abdus Sabur, Ishraque Karim, Aninda Nafis Ahmed

**Affiliations:** a Pilot Plant and Process Development Centre, Bangladesh Council of Scientific and Industrial Research (BCSIR) Dhanmondi Dhaka 1205 Bangladesh sabur37971@gmail.com; b Department of Materials Science & Engineering, Rajshahi University of Engineering & Technology (RUET) Rajshahi 6204 Bangladesh

## Abstract

The presence of toxic organic pollutants such as methyl orange (MO) and 4-nitrophenol (4-NP) in industrial effluents poses significant environmental and public health risks. This study reports a rapid, scalable, and eco-friendly microwave-assisted synthesis of gold nanoparticles (AuNPs) using *Azadirachta indica* leaf extract as a dual reducing-stabilizing agent. The synthesis parameters, including microwave power, irradiation time, and precursor concentration, were optimized to maximize catalytic activity. Comprehensive characterization *via* XRD, FTIR, TEM, FESEM, DLS, and UV-Vis spectroscopy confirmed the formation of crystalline, uniformly dispersed AuNPs (average size 11.90 ± 2.84 nm) exhibiting a distinct plasmonic resonance at 535 nm. A key novelty of this work is the direct comparison of biogenic AuNPs with commercial AuNPs for the NaBH_4_-mediated reduction of MO and 4-NP, monitored by UV-Vis spectroscopy under identical conditions. Green-synthesized AuNPs achieved about 75% MO and 70% 4-NP catalytic reduction within 10 minutes, with apparent rate constants of 0.059 ± 0.002 min^−1^ and 0.133 ± 0.066 min^−1^, respectively. The superior performance is attributed to a synergistic effect, where phytochemical capping agents from *Azadirachta indica* enhance pollutant adsorption and electron transfer between NaBH_4_ and the pollutant molecules *via* the AuNP core. Kinetic studies confirmed pseudo-first-order behavior. The proposed catalytic reduction mechanism involves efficient electron shuttling from BH_4_^−^ to pollutant molecules mediated by AuNPs, with bio-organic ligands acting as active surface sites. This comparative and mechanistic approach establishes a sustainable nanocatalyst platform for reducing hazardous organic contaminants and offers practical applicability for wastewater treatment.

## Abstract 2

The presence of toxic organic pollutants such as methyl orange (MO) and 4-nitrophenol (4-NP) in industrial effluents poses significant environmental and public health risks.

## Graphical abstract

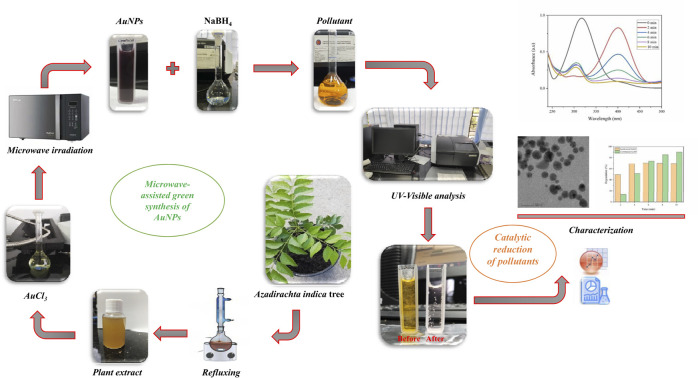

## 1 Introduction

The rapid expansion of industrial and urban activities has led to the widespread discharge of hazardous organic pollutants into aquatic ecosystems, with azo dyes and phenolic compounds being among the most persistent and toxic.^1–4^ Methyl orange (MO), widely used in textile and printing industries, is highly resistant to biodegradation due to its azo (–N=N–) linkages, while 4-nitrophenol (4-NP), a common by-product in agrochemical and pharmaceutical manufacturing, has been listed as a priority pollutant by the US Environmental Protection Agency (EPA) because of its toxicity, persistence, and bioaccumulative potential.^5–8^ Their accumulation in water bodies disrupts ecological balance and poses serious health risks, including carcinogenicity and organ toxicity.^7,9,10^

Conventional treatment techniques such as adsorption, coagulation, membrane filtration, photocatalysis, and biodegradation often suffer from high cost, secondary pollution, or limited efficiency against such recalcitrant pollutants.^11–14^ In contrast, catalytic reduction using noble metal nanoparticles, particularly gold nanoparticles (AuNPs), has emerged as a promising strategy due to their excellent electron mediation properties, high surface-to-volume ratio, and surface plasmon resonance (SPR) activity.^15–17^ In the presence of sodium borohydride (NaBH_4_), AuNPs accelerate electron transfer to pollutants, enabling rapid and efficient catalytic reduction under mild conditions.^18–20^

While chemically synthesized AuNPs are effective, they typically involve toxic reducing agents and solvents that raise environmental concerns. To overcome these limitations, green synthesis using plant extracts has gained significant attention as an eco-friendly, scalable, and sustainable approach.^21^*Azadirachta indica* (Neem) is particularly attractive because its phytochemicals-flavonoids, terpenoids, tannins, and phenolics-serve as dual reducing and stabilizing agents, promoting the formation of stable, biocompatible nanoparticles.^22^ Furthermore, microwave-assisted synthesis offers rapid heating, uniform nucleation, and improved control over nanoparticle properties, making it more efficient than conventional methods.^23,24^

Several studies have explored plant-mediated and microwave-assisted synthesis of AuNPs for pollutant's catalytic reduction.^18–20,25–27^ However, many of these approaches face limitations such as longer synthesis times, inconsistent nanoparticle stability, and incomplete pollutant's catalytic reduction. More importantly, despite the individual popularity of neem-based green synthesis and microwave-assisted methods, their combined application remains underexplored, and systematic comparisons with commercial AuNPs are scarce.

Table 1 presents a critical comparison of recent green and microwave-assisted AuNPs syntheses and their catalytic performance in pollutant's catalytic reduction, highlighting their synthesis strategy, catalytic metrics, and unresolved limitations.

**Table 1:** Critical comparison of recent green and microwave-assisted AuNPs syntheses and their catalytic performance in pollutant's catalytic reduction

Study (Year)	Plant/Method	Pollutant(s) degraded	Key findings	Limitations/gaps identified
S. Ahmad *et al.* (2024)^28^	*Aconitum violaceum* extract (green synthesis)	MO, 4-NP	Demonstrated catalytic reduction using NaBH_4_ in UV-Vis assay	Lack of systematic optimization, no comparison with commercial AuNPs
G. K. Deokar & A. G. Ingale (2023)^29^	Green-synthesized AuNPs	MO, RhB, CR	Showed effective catalytic reduction of dyes, including MO, with pseudo-first-order kinetics	Did not explore microwave-assisted synthesis or comparative commercial AuNPs
A. Bano *et al.* (2023)^30^	Citrate-synthesized AuNPs (chemical method)	Standard redox systems	Demonstrated size dependence of *k* in AuNP catalysis	Not a green synthesis; no phytochemical synergy investigated
L. Wang *et al.* (2023)^19^	Gold-phycoerythrin nanoparticles (algal biomolecule)	4-NP, cationic dyes	Reported an efficient, eco-friendly catalytic route for dye reduction	No microwave-assisted synthesis or comparison with commercial particles
T. L. Phan *et al.* (2025)^31^	Microwave-assisted embedding of AuNPs in carbon spheres	Environmental pollutants	Demonstrated a continuous microwave-assisted AuNPs process	Not focused on classic pollutant dyes; no phytochemical extract alone
This study	Microwave-assisted green synthesis of AuNPs using *Azadirachta indica* extract	MO, 4-NP	High activity comparable to commercial AuNPs	Novelty: First comparative study + synergistic mechanism explained

Despite these advances, gaps remain in terms of systematic microwave parameter optimization, synergistic mechanistic insight, and direct comparison against commercial AuNPs. The present study addresses these gaps by developing a microwave-assisted green synthesis of AuNPs using *Azadirachta indica* extract, followed by systematic optimization of microwave power, irradiation time, and precursor concentration. The structural, morphological, and optical properties of the synthesized nanoparticles are characterized using multiple techniques, including X-ray diffraction (XRD), Fourier-transform infrared spectroscopy (FTIR), field emission-scanning electron microscopy (FE-SEM), transmission electron microscopy (TEM), dynamic light scattering (DLS), and UV-Visible spectroscopy (UV-Vis). The novelty of this work lies in developing a microwave-assisted green synthesis route for AuNPs using *Azadirachta indica* extract, followed by a direct comparative evaluation of their catalytic efficiency against commercial AuNPs for the catalytic reduction of MO and 4-NP. Beyond assessing catalytic reduction efficiency, this study also highlights the synergistic role of AuNPs, NaBH_4_, and phytochemical capping agents in enhancing pollutant adsorption and electron transfer. This dual emphasis on comparative analysis and mechanistic understanding offers new insights into the sustainable design of nanocatalysts for wastewater treatment.

## 2 Experimental details

### 2.1 Materials

Commercial gold nanoparticles (20 nm diameter, stabilized suspension in citrate buffer), gold(iii) chloride, 4-nitrophenol, and sodium borohydride were purchased from Sigma-Aldrich. Methyl orange was purchased from Merck KGaA. All chemicals were used as received without any further purification. Ethanol and distilled water were also purchased from Sigma-Aldrich and were used for the preparation of all aqueous solutions.

### 2.2 Methods

#### 2.2.1 Preparation of *Azadirachta indica* extract

**Fig. 1:**
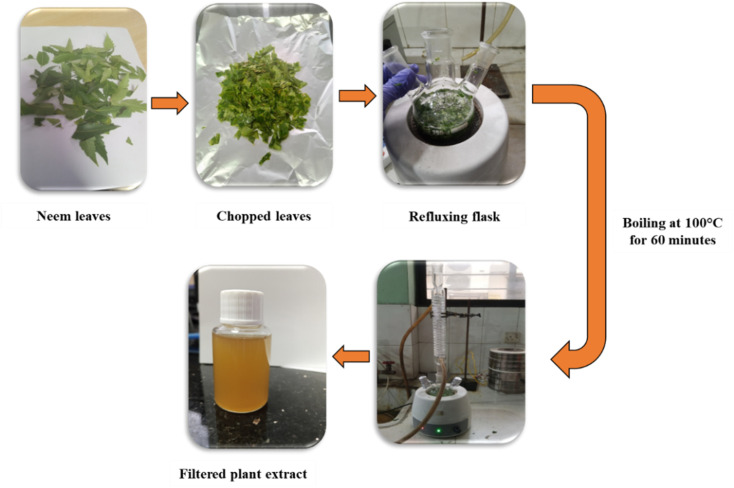
Schematic illustration of the preparation of *Azadirachta indica* leaf extract used for green synthesis.

Fig. 1 shows the overall process for the preparation of *Azadirachta indica* extract. Fresh, mature *Azadirachta indica* leaves were collected from a healthy tree located inside the Bangladesh Council of Scientific and Industrial Research (BCSIR), Dhaka, Bangladesh. The plant species was identified and authenticated based on standard morphological characteristics consistent with *Azadirachta indica* as described in regional floras. The collected leaves were thoroughly washed with distilled water to remove dust and air-dried at room temperature (25 °C). A pair of scissors was used to chop the dried leaves, from which 25 g of leaves were then transferred to a refluxing flask containing 100 mL of distilled water. The mixture was subjected to extraction at 100 °C for 60 minutes, and a reflux condenser was used to ensure continuous boiling without loss of solvent. The extracted material was cooled to room temperature, filtered, and then stored at 4 °C for further use in the synthesis of Au nanoparticles. We repeated these steps, maintaining 1 : 4 leaf-to-solvent ratios to produce three batches of plant extract, which were further analyzed by FTIR, which gave consistent functional groups.

#### 2.2.2 Microwave-assisted green synthesis of Au nanoparticles using *Azadirachta indica* extract

**Fig. 2:**
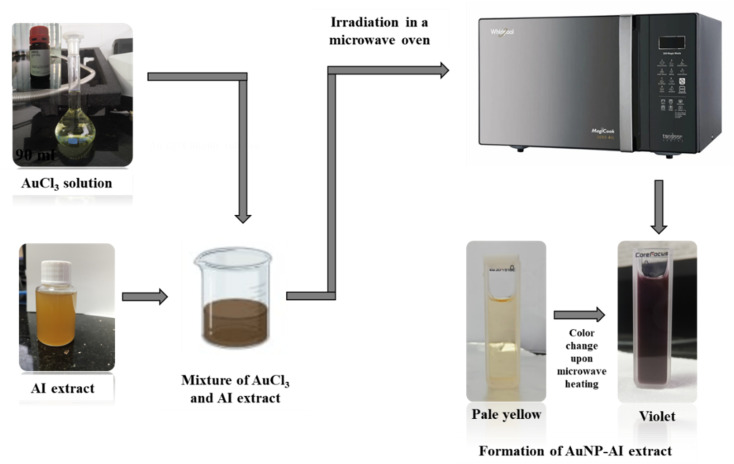
Schematic representation of the microwave-assisted green synthesis of gold nanoparticles using *Azadirachta indica* leaf extract.

10 mL of *Azadirachta indica* extract was mixed with 90 mL of aqueous gold(iii) chloride solutions at different concentration levels of 0.5 mM, 0.75 mM, and 1 mM. Each mixture was subjected to microwave irradiation for 20, 40, and 60 seconds using a domestic microwave oven operating at different power levels of 450 W, 720 W, and 900 W. The formation of AuNPs was initially indicated by a visible color change from pale yellow to violet upon heating in a microwave oven, which confirms the reduction of Au^3+^ ions to Au^0^ nanoparticles mediated by the AI plant extract. This was also further confirmed by the UV-visible spectra. A schematic diagram of this process is illustrated in Fig. 2. Control tests demonstrated that no AuNPs formation occurred in precursor solutions stored without plant extract, confirming the essential role of phytochemicals as reducing and stabilizing agents. Likewise, synthesis carried out without microwave irradiation produced only a weak and delayed color change, indicating that microwave heating dramatically accelerates the reduction and nucleation process. Together, these observations establish the synergistic contributions of both the plant extract and microwave irradiation in the successful synthesis of AuNPs.

#### 2.2.3 Catalytic study of methyl orange and 4-nitrophenol

**Fig. 3:**
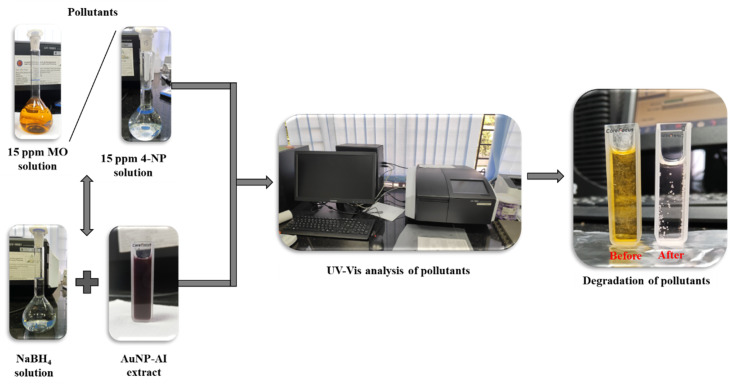
Schematic diagram illustrating the catalytic reduction setup and reaction pathway for methyl orange and 4-nitrophenol using AuNPs.

Both methyl orange and 4-nitrophenol were prepared individually at concentrations of 10 ppm and 15 ppm. For the study of each catalytic reduction, 2 mL of the pollutant solution was mixed with freshly prepared 0.75 mL 20 mM NaBH_4_ and 0.25 mL 1 mM of Au nanocatalyst in a quartz cuvette (path length:10 mm). The catalytic reduction progress was monitored using UV-Vis spectroscopy at 2 minute intervals in the 200–800 nm wavelength range (Fig. 3).

The percentage of catalytic reduction was determined as follows:1
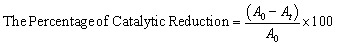
where *A*_0_ indicates the initial absorption of the dye solution, and *A*_*t*_ indicates the final absorption of the dye at specified time intervals.

To ensure a fair and consistent comparison, all catalytic reduction experiments using green-synthesized AuNPs and commercial AuNPs were performed under identical operational conditions. The same reaction volume, pollutant concentration (2 mL), NaBH_4_ volume (0.75 mL), AuNP suspension volume (0.25 mL), optical path length (10 mm quartz cuvette), temperature, and UV-Vis monitoring conditions were maintained throughout. While the commercial AuNPs were citrate-stabilized with an average particle size of ∼20 nm and the green-synthesized AuNPs exhibited a smaller core size (11.90 nm by TEM) with a broader hydrodynamic diameter due to phytochemical capping, these differences are intrinsic to the synthesis routes and were intentionally preserved to evaluate their influence on catalytic performance.

### 2.3 Characterization

The synthesized AuNPs were characterized using a combination of techniques to confirm their structural, morphological, and optical properties. X-ray diffraction (XRD) was used to determine crystallinity, crystallite size, and phase identification, confirming the face-centered cubic (FCC) structure of gold. Transmission electron microscopy (TEM), including high-resolution TEM (HRTEM) and selected area electron diffraction (SAED), provided information on particle morphology, size distribution, and lattice fringes. Field emission scanning electron microscopy (FESEM) was employed to examine surface morphology and particle dispersion, while energy-dispersive X-ray spectroscopy (EDS) verified the elemental composition and distribution of gold and associated phytochemicals. Fourier transform infrared (FTIR) spectroscopy was conducted to identify functional groups in the *Azadirachta indica* extract responsible for reducing and stabilizing the nanoparticles. Dynamic light scattering (DLS) was used to assess the hydrodynamic size and distribution of the AuNPs in aqueous suspension. Finally, UV-Visible spectroscopy (UV-Vis) was applied to study the optical properties, particularly surface plasmon resonance (SPR), to monitor the influence of synthesis conditions on nanoparticle formation, and to investigate the catalytic reduction of organic pollutants.

## 3 Results and discussion

### 3.1 XRD analysis

**Fig. 4:**
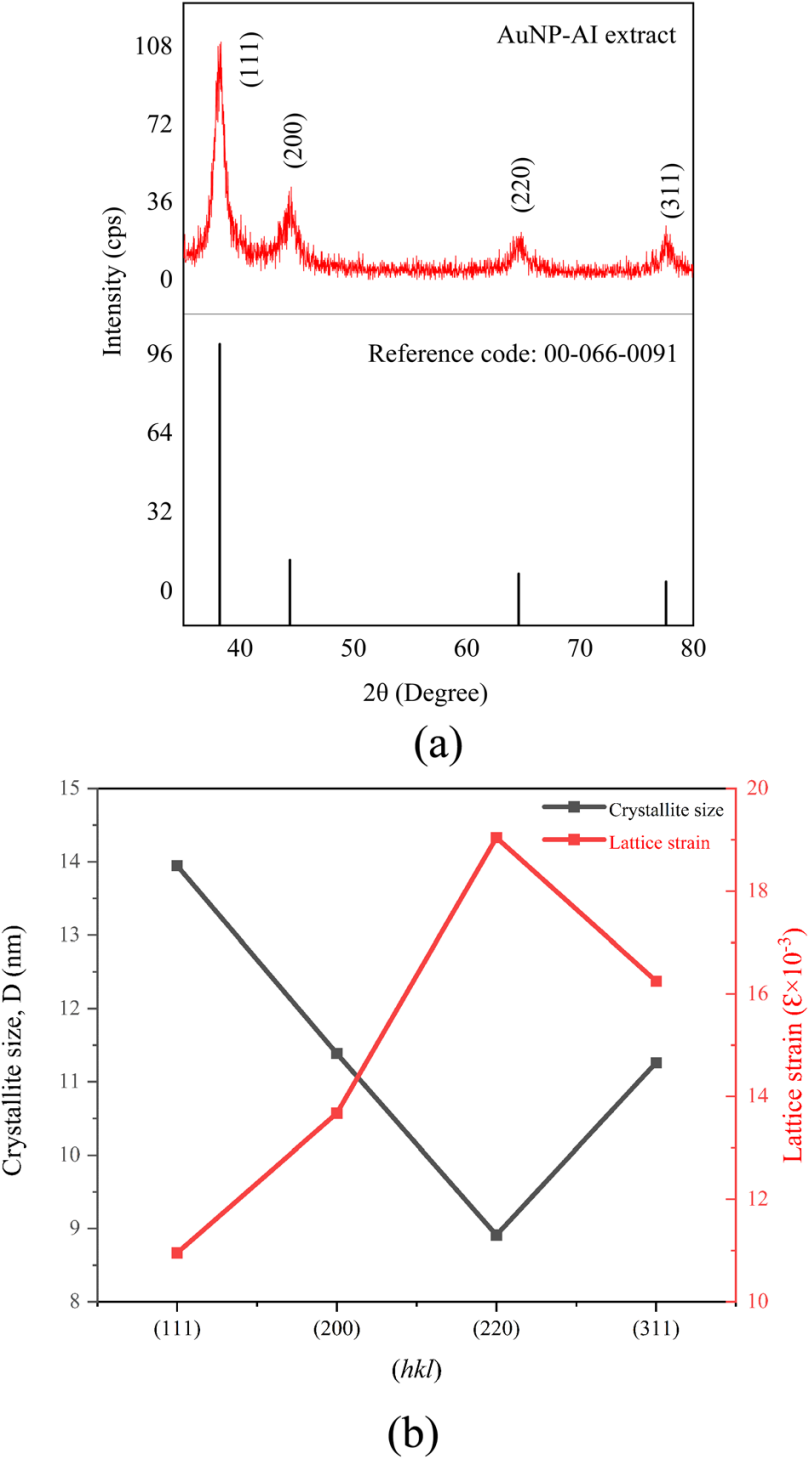
(a) XRD pattern of green-synthesized AuNP-AI, confirming crystalline gold formation; (b) variation of crystallite size and lattice strain of AuNP-AI.

The crystal structure, orientation, and phase purity of AuNP-AI extract were analyzed by using X-ray diffraction analysis. Fig. 4a represents the XRD pattern of Au nanoparticles synthesized *via* microwave-assisted green route from *Azadirachta indica*. The AuNP-AI exhibits a polycrystalline nature with diffraction peaks located at 38.30°, 44.45°, 64.70°, and 77.46° associated with the (*hkl*) planes of (111), (200), (220), and (311), respectively. All the observed diffraction peaks have a single phase, and no additional peaks were observed. The synthesized Au nanoparticles exhibited a face-centered cubic (FCC) structure, and the XRD spectrum matched the International Center for Diffraction Data (ICDD) reference code: 00-066-0091.^32,33^

A preferential growth in the diffraction pattern is observed along the (111) plane, where the peak is intense and prominent, and the strength of preferential growth along the (111) plane is so large compared to the (200), (220), and (311) planes. This preferential orientation can influence the physical and chemical properties of the Au nanoparticles, which are suitable for a stable crystalline structure and desirable catalysis applications.^34^

The peak intensity is significantly lower along the (200) plane compared to the (111) plane, and this reduced peak intensity along the (200) plane indicates that fewer Au atoms are oriented along this direction, and the (200) plane has lower crystallographic stability compared to the (111) plane.^35^ The synthesized Au nanoparticles also have a higher strain (13.674 × 10^−3^) value along the (200) plane compared to the (111) plane (Table 2), which also indicates the lower stability and lower preferential growth along the (200) plane.

**Table 2:** Structural parameters of green-synthesized gold nanoparticles derived from XRD analysis

Sample	*hkl*	2*θ* (degree)	*d*-spacing [Å]	FWHM [degree]	Lattice parameter, *a* (Å)	Crystallite size, *D* (nm)	Dislocation density, *δ* × 10^−3^ (nm^−2^)	Lattice strain, *ε* × 10^−3^	Volume of unit cell, Å^3^
AuNP-AI extract	(111)	38.30	2.348	0.629	4.07	13.946	5.141	10.953	67.39
	(200)	44.45	2.036	0.787	4.07	11.386	7.712	13.674	67.64
	(220)	64.70	1.439	1.102	4.07	8.912	12.590	19.043	67.32
	(311)	77.46	1.231	0.944	4.08	11.260	7.886	16.251	68.26

The crystallite size, *D* of AuNP-AI extract, was calculated using Scherrer's formula.^36^2
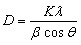
where *β* is the full width at half-maximum (FWHM) of the corresponding peak, *θ* is Bragg's angle, *λ* is the X-ray wavelength, and *K* is the shape factor. Fig. 4b illustrates the variation of the crystallite size of AuNP-AI extract as a function of different crystallographic planes. The crystallite sizes of 13.946 nm, 11.386 nm, 8.912 nm, and 11.26 nm were observed along the (111), (200), (220), and (311) planes, respectively. The crystallite size decreased along the (220) plane compared to the others, as the (220) plane possesses a higher surface energy than the (111) plane, which impedes the extensive growth and results in smaller crystallite sizes.^37^ The largest crystallite size was observed along the (111) plane, as this plane has the lowest strain (10.953 × 10^−3^) compared to other crystallographic planes (Table 2).

The lattice parameter (*a*), the volume of the unit cell (*V*), lattice strain (*ε*), and the dislocation density (*δ*) were measured using the following equations^38^3
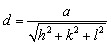

4 *V* = *a*^3^

5
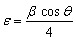
6
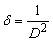


Higher strain values were observed for the (200), (220), and (311) planes compared to the (111) plane, as shown in Table 2. The green synthesis process of Au nanoparticles using *Azadirachta indica* extract introduces some reducing and capping biomolecules (*e.g.*, flavonoids, terpenoids), which tend to adsorb selectively on the Au nanoparticle surfaces and make (200), (220), and (311) planes less stabilized compared to the (111) plane and induce differential strain along these planes.^39,40^

The lowest dislocation density, 5.141 × 10^−3^ nm^−2^, was observed for the (111) plane, and the dislocation density increased up to 12.590 × 10^−3^ nm^−2^ for the (220) plane. As the coherent crystallite size along the (111) plane is greater than that along the (200), (220), and (311) planes (Table 2), this anisotropic behavior is responsible for a lower dislocation density for the (111) plane and a higher dislocation density for the other planes.

### 3.2 TEM analysis

**Fig. 5:**
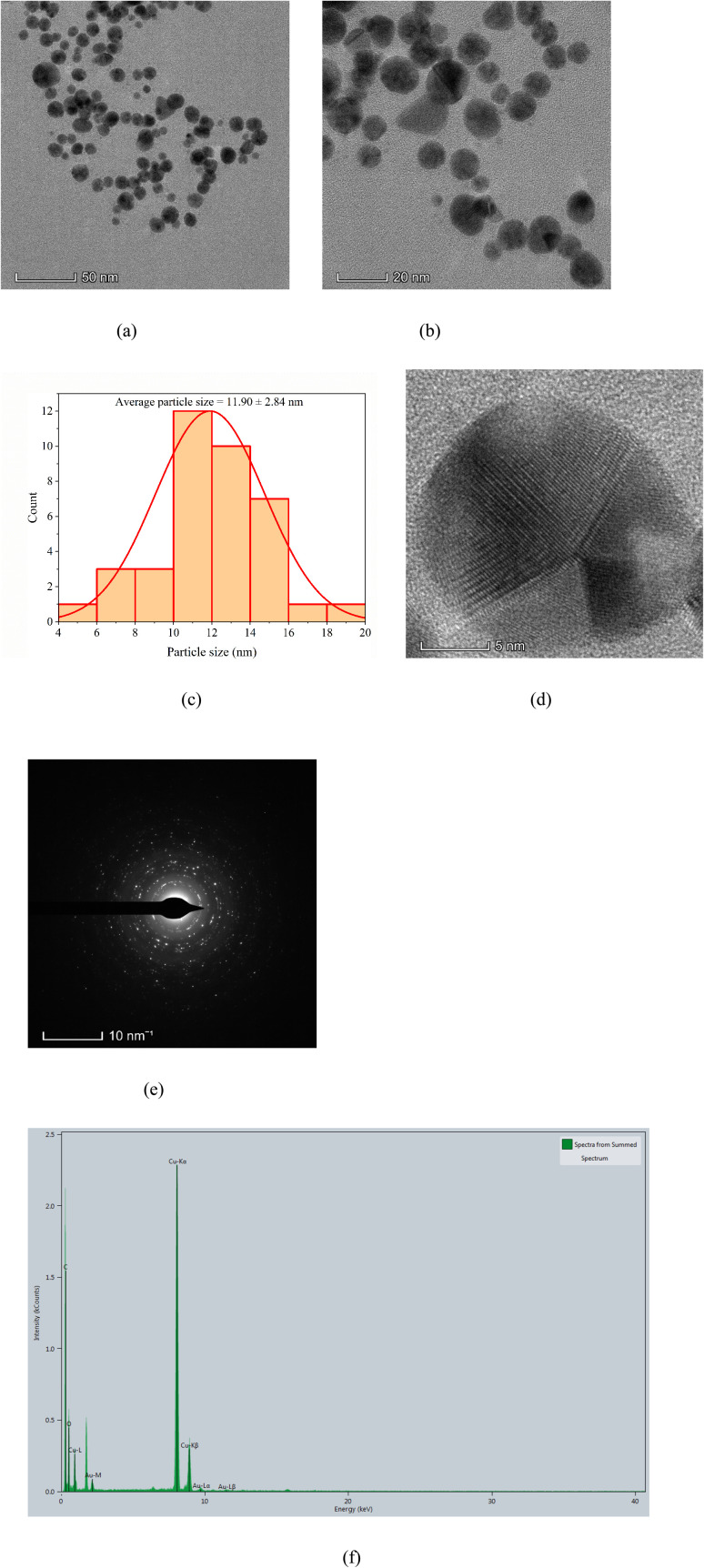
(a and b) TEM images of green-synthesized AuNP-AI at 50 nm and 20 nm scale bars, showing well-dispersed, quasi-spherical nanoparticles; (c) corresponding particle size distribution histogram; (d) HRTEM image highlighting lattice fringes of crystalline AuNPs; (e) SAED pattern confirming the face-centered cubic (fcc) crystalline structure of gold; and (f) EDS spectrum verifying the elemental composition of AuNP-AI.

TEM analysis was conducted to evaluate the size, shape, and dispersion of the gold nanoparticles synthesized using *Azadirachta indica* extract under optimized microwave-assisted conditions. The TEM micrographs in Fig. 5a and b demonstrate that the AuNPs are predominantly spherical, with a moderate occurrence of quasi-spherical and truncated shapes. The nanoparticles are well-dispersed, indicating effective stabilization by the phytochemicals in the AI extract, which act as both reducing and capping agents during synthesis. Such morphologies are typical in biogenically synthesized AuNPs and have been observed in similar studies using plant extracts.^41–43^

The particle size distribution histogram in Fig. 5c indicates that most particles are between 10 and 14 nm, with an average particle diameter of 11.90 ± 2.84 nm. This size range is ideal for catalytic activity, offering a high surface-to-volume ratio while minimizing the risk of aggregation. Previous research also indicates that AuNPs between 10 and 30 nm exhibit improved catalytic performance in redox reactions.^44^

High-resolution TEM (HRTEM) imaging in Fig. 5d confirms the crystalline nature of the AuNPs, with clearly visible lattice fringes and an interplanar spacing consistent with the (111) planes of face-centered cubic gold. This crystalline structure is further supported by the selected area electron diffraction (SAED) pattern in Fig. 5e, which shows distinct diffraction rings indexed to the (111), (200), (220), and (311) planes. This pattern verifies the polycrystalline nature of the synthesized AuNPs and aligns well with the standard FCC gold structure (ICDD reference code: 00-066-0091), as also noted in XRD studies.^32^

Energy-dispersive X-ray (EDX) spectroscopy in Fig. 5f confirms the elemental composition of the nanoparticles, showing strong signals at approximately 2.1 keV and 9.7 keV that correspond to gold. Sharp peaks for copper are observed due to the use of a copper TEM grid. The presence of carbon and oxygen is due to phytochemicals in the plant extract, which act as natural reducing and stabilizing agents on the surfaces of the AuNPs.

### 3.3 FESEM and EDS analysis

#### 3.3.1 FESEM analysis

**Fig. 6:**
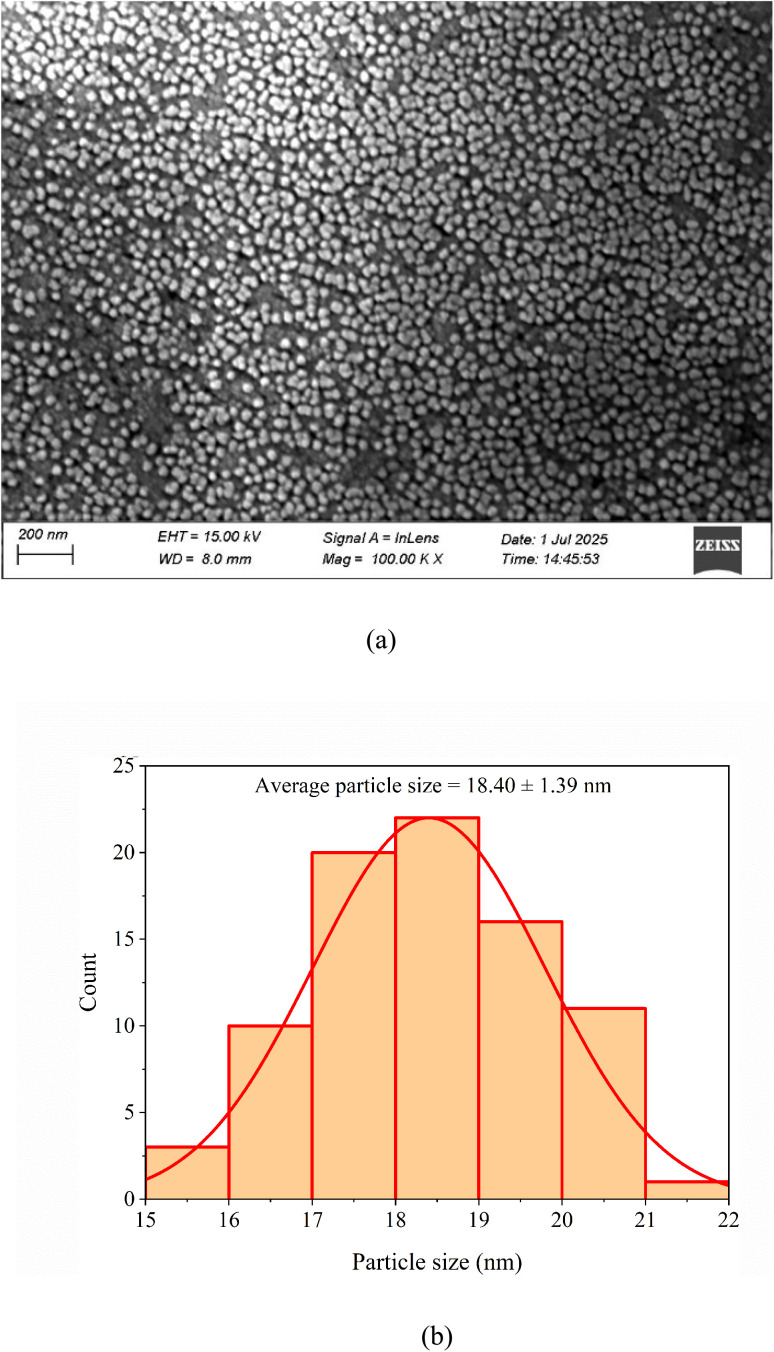
(a) FESEM micrograph showing the surface morphology of green-synthesized AuNPs, and (b) corresponding particle size distribution derived from FESEM analysis.

Fig. 6a represents the FESEM image of Au nanoparticles synthesized *via* a microwave-assisted green route from *Azadirachta indica*. The obtained FESEM image of AuNPs reveals the well-isolated and uniformly distributed, nearly spherical to quasi-spherical shaped grains, which suggests the effective nucleation and controlled growth during the green synthesis process. Each bright grain in the FESEM image of AuNP-AI extract represents a crystalline AuNP.

The bright contrast and sharp edges indicate the well-defined morphology of AuNP-AI extract, the good metallic nature of Au nanoparticles, and surface smoothness. The uniform contrast also suggests the homogeneity in particle size and surface composition, which facilitates the stabilization of bioactive compounds in the AI extract.

Some active bio-reductant compounds in AI leaves (flavonoids and terpenoids) facilitate the Au ions from soluble precursors into metallic gold nanoparticles through a reduction reaction.^45^ The flavonoid compounds in AI leaves can significantly enhance the stability and size control of synthesized AuNP-AI extract due to their binding ability to AuNP surfaces.^45^ These types of bioactive compounds in AI prevent the particles from agglomeration, leading to more stable nanoparticles.^46^ In this study, the Au nanoparticles also appear to be well-separated with minimal agglomeration.

Fig. 6b shows the particle size distribution from FESEM analysis. The histogram exhibits a Gaussian-like shape, with most particles between 17 and 20 nm and an average size of 18.40 ± 1.39 nm, indicating a narrow, unimodal distribution. This size range aligns with other biogenically synthesized AuNPs and is considered ideal for applications needing strong surface plasmon resonance and high catalytic efficiency.^44^ The size control demonstrated here supports findings from TEM analysis, confirming the effectiveness of the microwave-assisted green synthesis method.

#### 3.3.2 EDS analysis

**Fig. 7:**
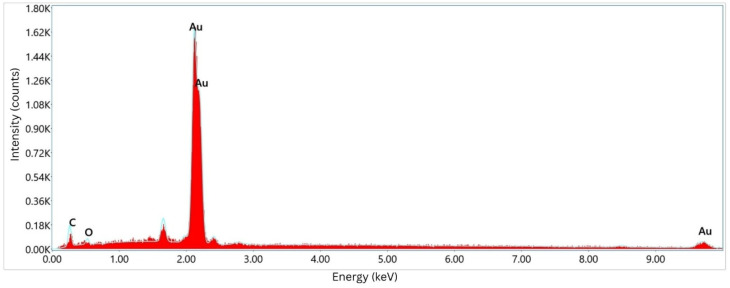
EDS spectrum of AuNP-AI confirming the elemental composition of the green-synthesized gold nanoparticles.

EDS analysis was performed to determine the elemental composition of gold nanoparticles synthesized from *Azadirachta indica* leaf extract. Fig. 7 shows the EDS spectrum of AuNP-AI extract, confirming the successful formation of Au nanoparticles by the presence of a sharp peak corresponding to elemental Au around 2.2 keV, which is the M-shell ionization of Au.

The minor peaks for C and O were also observed at approximately 0.72 keV and 0.52 keV, respectively. The presence of C and O spectra indicates the possible capping of AuNPs by phytochemicals such as flavonoids, terpenoids, and other organic constituents. These biomolecules can be adsorbed onto the forming nanoparticle surface, resulting in organic residues, which are subsequently detected as C and O in the EDS spectrum.^47,48^

The quantitative EDS data of the AuNP-AI extract in Table 3 reveal that the Au elements comprise the majority of the sample, with a weight percentage of 91.0%, while C and O contribute 7.1% and 1.9%, respectively. The high amount of Au content in the EDS spectrum of AuNP-AI extract indicates the successful reduction of Au^3+^ ions to Au^0^ by the *Azadirachta indica* extract during the green synthesis process.^49^

**Table 3:** Elemental composition of AuNP-AI determined by FESEM-EDS analysis

Element	Weight %	MDL	Atomic %	Net int	Error %	*R*
C K	7.1	2.77	50.3	13.2	27.4	0.6544
O K	1.9	0.83	10.1	11.1	27.8	0.6661
Au M	91.0	1.13	39.5	665.6	6.2	0.7185

The net intensity of 665.6 and the low error percentage (6.2%) for gold further confirm its prominent presence in the nanoparticle structure. The relatively high atomic percentage of carbon (50.3%) indicates significant organic content associated with the nanoparticles, which is common in green-synthesized AuNPs due to the bio-reducing environment.^50,51^ This also confirms the successful biofunctionalization of AuNPs, enhancing colloidal stability and surface reactivity, which are key attributes for catalytic and biomedical applications.^52,53^

#### 3.3.3 Elemental mapping of AuNP-AI extract

**Fig. 8:**
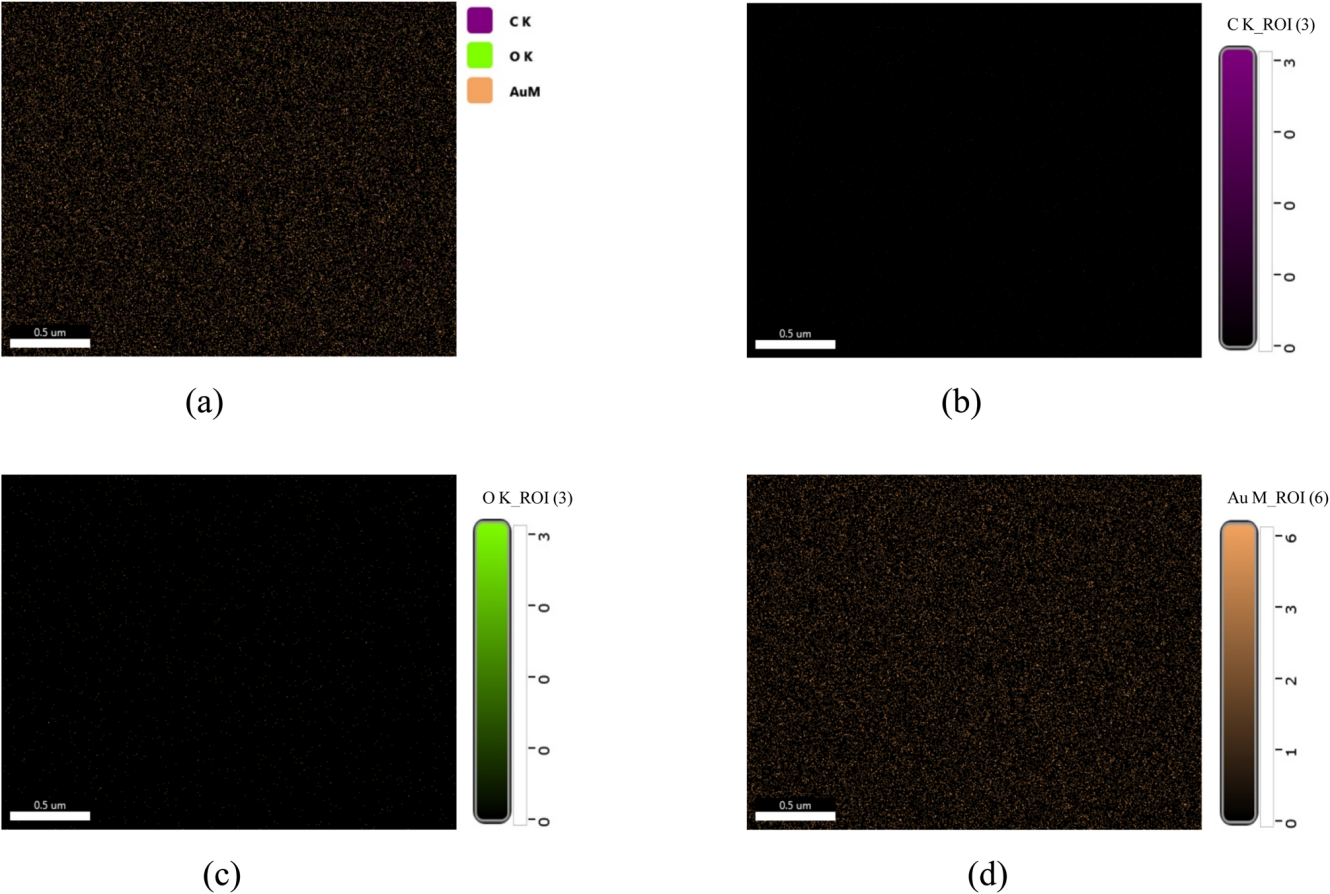
(a) SEM-EDS elemental mapping of AuNP-AI, and spatial distribution of (b) carbon (C), (c) oxygen (O), and (d) gold (Au), demonstrating uniform elemental dispersion within the nanoparticles.

The elemental distribution of AuNP-AI extract was evaluated using EDS mapping integrated with FESEM. Fig. 8a illustrates the elemental mapping images and demonstrates the spatial distribution of C, O, and Au within the scanned area of Au nanoparticles synthesized from *Azadirachta indica* leaf extract.

The elemental overlay map clearly shows the co-existence of C, O, and Au nanoparticles. Carbon (C K) is shown in purple color in the image, which originates from the organic compounds present in the AI extract. Oxygen (O K) appears in green color. Some phytochemicals, including flavonoids and terpenoids in AI extract, can undergo oxidation during the nanoparticle synthesis process, and this oxidation may form oxygenated compounds that bind to the AuNPs surface.^54^ Au nanoparticles are shown in orange color, which represents the successful formation and dispersion of AuNP across the substrate. The dense and uniform orange coloration across the sample proves the homogeneous distribution of AuNPs throughout the scanned region.

Fig. 8b represents the map of Carbon (C K), where a faint and almost negligible distribution of C is observed. The low intensity of C signals confirms the lower organic contamination on the surface during the nanoparticle's synthesis process. The oxygen (O K) map (Fig. 8c) reveals a mild but visible distribution of O, which is attributed to the oxygenated biomolecules from AI extract.^54^

Fig. 8d represents the elemental mapping of gold (Au M) that appears as a strong and uniform distribution of gold signals, which confirms the successful formation and wide dispersion of Au nanoparticles. The high-intensity signals of Au throughout the area confirm the abundant presence of Au in the sample and ensure the efficient bio-reduction of Au^3+^ ions into elemental Au.

### 3.4 FTIR analysis

**Fig. 9:**
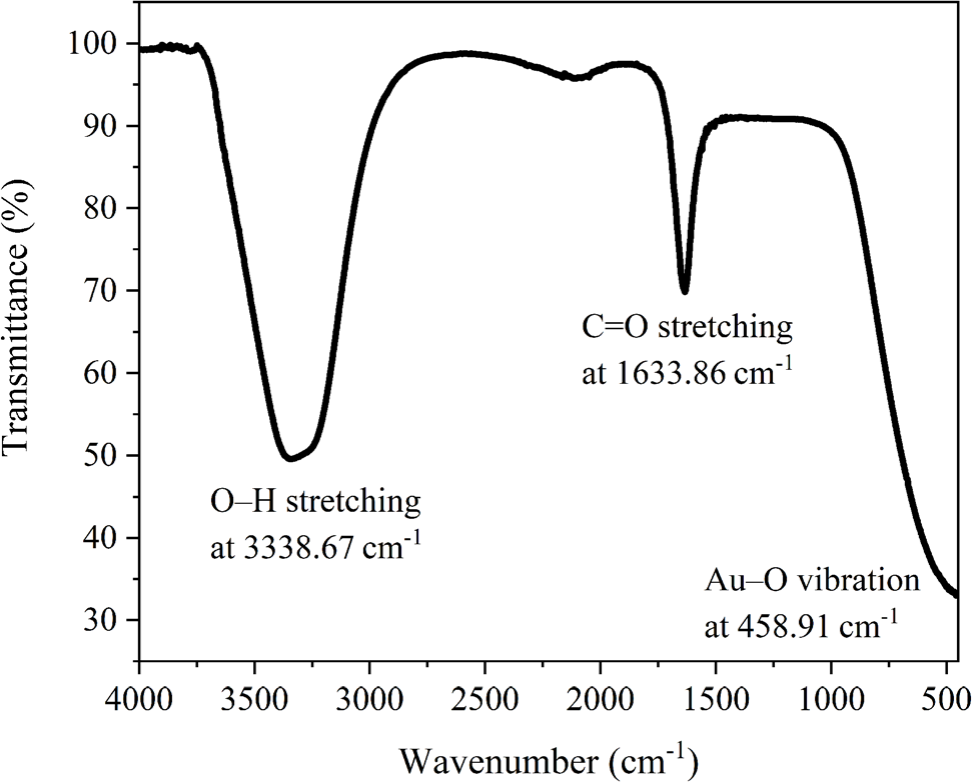
FTIR spectra of green-synthesized AuNP-AI, illustrating the functional groups involved in nanoparticle reduction and surface stabilization.

The FTIR spectrum of AuNP-AI extract (Fig. 9) shows characteristic peaks at 3338.67 cm^−1^, 1633.86 cm^−1^, and 458.91 cm^−1^, indicating the involvement of various phytochemicals in the reduction and stabilization processes.

The broad and intense absorption band at 3338.67 cm^−1^ corresponds to the O–H stretching vibration of hydroxyl groups found in polyphenols, flavonoids, and tannins.^55,56^ These compounds are recognized as natural reducing agents, helping convert Au^3+^ to elemental Au^0^ during nanoparticle synthesis.^57^ The persistence of this peak also indicates their adsorption onto the nanoparticle surface, aiding colloidal stability through hydrogen bonding and electrostatic repulsion.

A strong peak is observed at 1633.86 cm^−1^ corresponding to C=O stretching or aromatic C=C stretching in flavonoids, terpenoids, and other phenolic compounds.^58^ These groups also facilitate electron donation, enabling the reduction of gold ions and aiding in metal–ligand complex formation at the nanoparticle interface. The interaction between these groups and the nanoparticle surface causes slight peak shifts and intensity changes, supporting their role as capping agents.

A noteworthy low-frequency absorption at 458.91 cm^−1^ can be attributed to Au–O or Au–Cl bond vibrations, indicating the formation of metal–ligand bonds between gold and the biomolecules in *Azadirachta indica* extract.^59^ This peak is regarded as a signature of nanoparticle formation, confirming that phytochemicals not only reduce Au^3+^ but also bind to the metallic surface, ensuring dispersion and long-term stability.

### 3.5 DLS analysis

**Fig. 10:**
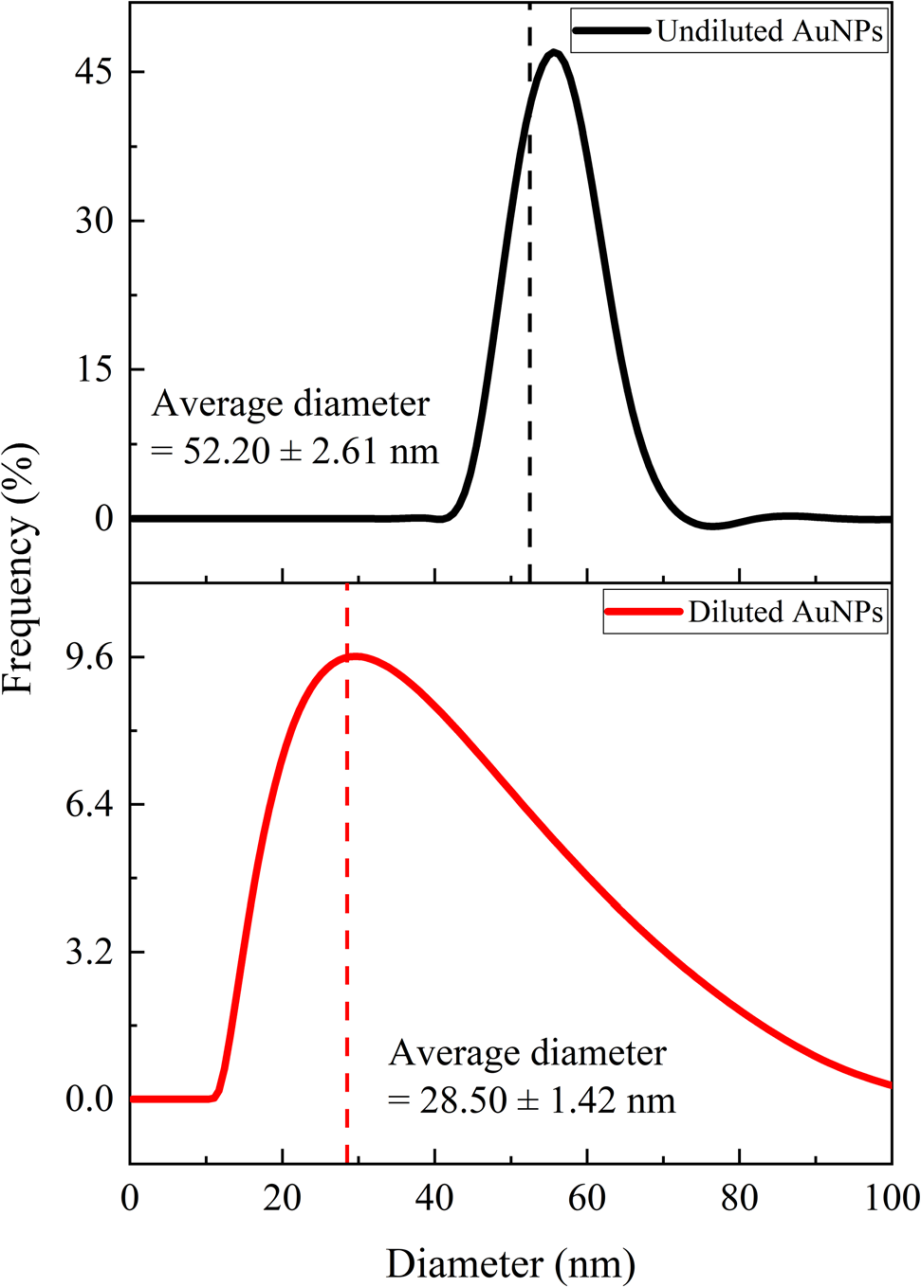
Dynamic light scattering analysis showing the hydrodynamic particle size distribution of AuNP-AI in aqueous suspension.

Fig. 10 illustrates the DLS analysis of the AuNP-AI extract, providing insights into nanoparticle size distribution and colloidal stability. For the undiluted suspension, the particles exhibited a mean hydrodynamic diameter of 52.20 ± 2.61 nm with a polydispersity index (PDI) of 0.388 ± 0.019. This relatively higher value reflects the influence of the phytochemical capping layer from *Azadirachta indica* extract and possible weak aggregation at higher nanoparticle concentrations. The DLS spectrum displayed a monomodal distribution with no significant secondary peaks, indicating that the colloid remained relatively stable.

To further confirm size and dispersion behavior, a diluted sample (dilution factor = 2) was analyzed, which showed a mean hydrodynamic diameter of 28.50 ± 1.42 nm and a markedly lower PDI of 0.173 ± 0.008. The low PDI (<0.2) suggests that the AuNPs are highly monodisperse and well-dispersed under diluted conditions, with minimal aggregation.^60^ These values are more consistent with the sizes observed from FESEM (∼18 nm) and TEM (∼12 nm) analyses, confirming that DLS measurements in concentrated suspensions tend to overestimate particle size due to contributions from the solvation shell and the organic capping layer.^61,62^

It is important to note that DLS measures the hydrodynamic diameter, which includes the metallic gold core, the surrounding bio-organic corona (flavonoids, terpenoids, phenolics, and tannins), and the associated hydration layer. Therefore, DLS-derived sizes are generally larger than those obtained from electron microscopy.^63,64^ The stability and monodispersity reflected in the low PDI values demonstrate effective nanoparticle stabilization by the phytochemicals in AI extract, which serve the dual function of reducing Au^3+^ to Au^0^ and preventing aggregation.^65^ Such uniformity is essential for catalytic applications, as it ensures consistent surface area and reproducible reactivity.^66,67^

### 3.6 UV-vis analysis

#### 3.6.1 UV-vis analysis of AuNP-AI extract

**Fig. 11:**
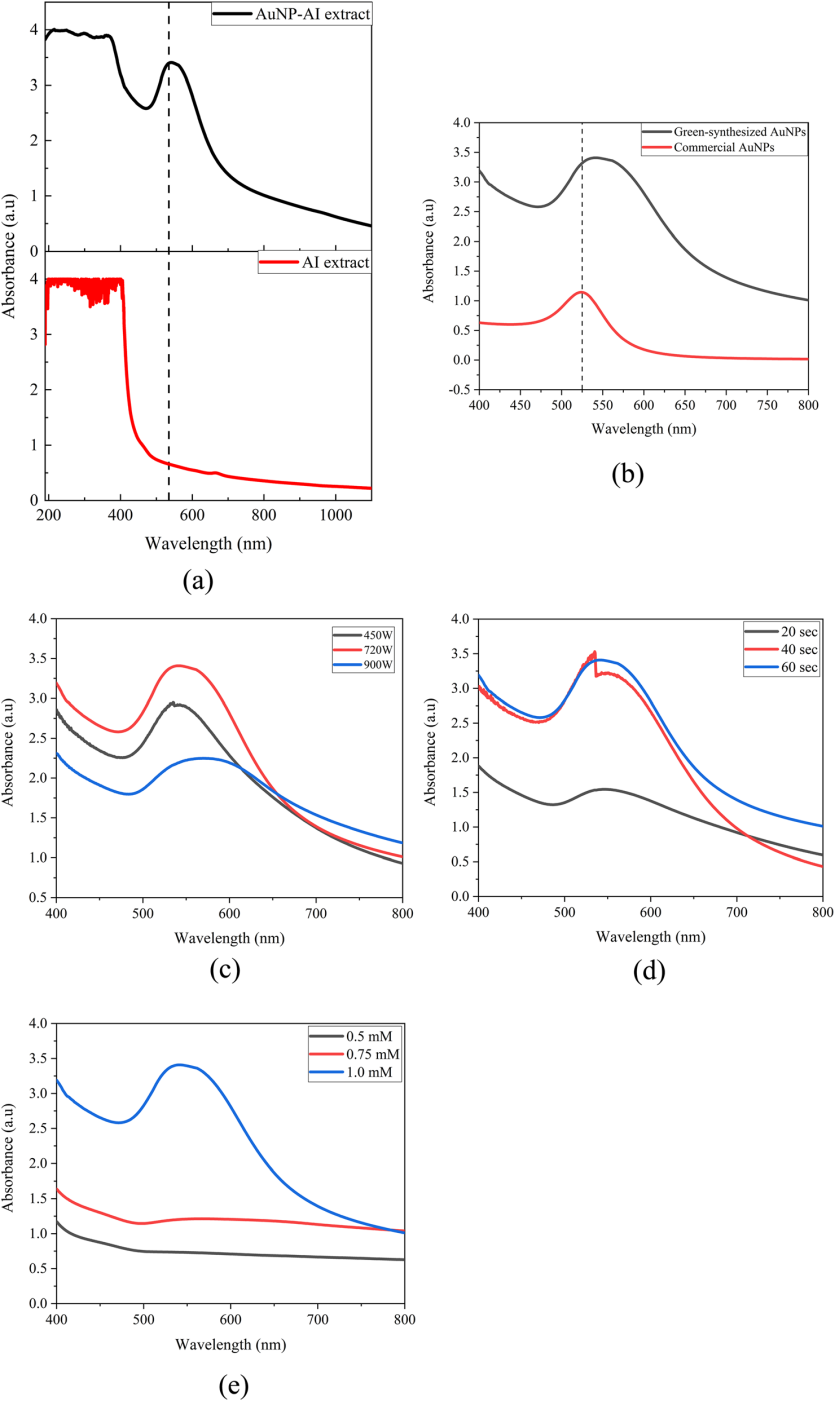
(a) UV-Vis spectra of *Azadirachta indica* extract and green-synthesized AuNP-AI, (b) comparison of UV-Vis spectra of green-synthesized and commercial AuNPs, (c) effect of microwave power on the UV-Vis spectra of AuNP-AI, (d) effect of irradiation time on the UV-Vis spectra of AuNP-AI, and (e) effect of Au^3+^ precursor concentration on the UV-Vis spectra of AuNP-AI.

UV-Visible spectroscopy was used to monitor and evaluate the formation and quality of AuNPs synthesized through microwave-assisted green synthesis using *Azadirachta indica* leaf extract. The comparative UV-Vis spectra (Fig. 11a) for the AI extract and the synthesized AuNP-AI sample show a strong surface plasmon resonance (SPR) peak at around 535 nm in the AuNP-AI spectrum, which is not present in the AI extract. This clear plasmonic band confirms the successful reduction of Au^3+^ to metallic Au^0^ and the formation of colloidal gold nanoparticles.^68^ The SPR band is typical of spherical or nearly spherical AuNPs and suggests nanoscale sizes.^44,69^ Compared to commercial AuNPs, the green-synthesized AuNPs exhibited a slight red shift of the SPR band (from ∼525 nm to ∼535 nm) along with noticeable peak broadening, as shown in Fig. 11b. This shift can be attributed to the smaller average particle size (∼11.9 nm) and the influence of phytochemical capping agents from *Azadirachta indica*, which alter the local dielectric environment around the AuNPs. The broadening indicates a relatively wider size distribution compared to the narrowly dispersed commercial AuNPs, consistent with the FESEM and DLS results. Similar spectral behavior has been reported for plant-mediated AuNPs in previous studies.^70^

To optimize nanoparticle synthesis, key parameters-microwave power, irradiation time, and precursor concentration were systematically varied and analyzed using UV-Vis spectroscopy (Fig. 11c–e). Among the tested conditions, the sample synthesized at 720 W for 60 seconds with 1 mM AuCl_3_ showed the most intense and sharp SPR peak, centered at 535 nm. This indicates a higher concentration of well-dispersed nanoparticles with uniform size distribution and minimal aggregation, as evidenced by the narrower peak bandwidth compared to other conditions.

Lower microwave power or shorter irradiation times (*e.g.*, 480 W or 30 s) resulted in broader, less intense SPR bands, indicating incomplete reduction or insufficient nucleation of AuNPs.^71^ Conversely, higher power or longer exposure (*e.g.*, 900 W or >60 s) likely caused overheating and possible aggregation, as shown by red-shifting or broadening of the SPR peak.^72^ Similarly, increasing the precursor concentration above 1 mM did not further improve nanoparticle quality and, in some cases, caused broader peaks due to excessive nucleation and agglomeration.^55^ At concentrations of 0.5 mM and 0.75 mM, the formation of gold nanoparticles is minimal because there aren't enough Au^3+^ ions for effective nucleation and growth. Surface plasmon resonance peaks are weak or absent, indicating incomplete reduction or the creation of very small clusters that do not exhibit the typical optical properties of AuNPs.^73,74^

To quantitatively optimize the microwave-assisted synthesis, the effects of microwave power, irradiation time, and precursor concentration were evaluated by comparing the SPR peak intensity, position, and bandwidth in the UV-Vis spectra (Fig. 11c–e). The condition of 720 W for 60 s with 1 mM AuCl_3_ produced the most intense and narrow SPR peak at ∼535 nm, indicating efficient reduction and formation of well-dispersed AuNPs. Lower power or shorter irradiation times resulted in weak, broadened SPR bands due to incomplete reduction, while excessive power led to peak broadening and red-shifting associated with aggregation. Based on these quantitative spectral trends, this condition was selected as optimal for subsequent characterization and catalytic studies.

Reproducibility of the optimized synthesis was evaluated by repeating the microwave-assisted reaction (720 W, 60 s, 1 mM AuCl_3_) using independently prepared batches of *Azadirachta indica* extract. All batches produced AuNPs with consistent surface plasmon resonance peaks centered at ∼535 nm, with comparable peak intensities and bandwidths. Subsequent characterization (TEM, DLS, and XRD) confirmed similar particle sizes, size distributions, and crystalline structures across batches. These results demonstrate good batch-to-batch reproducibility of the optimized green synthesis protocol.

#### 3.6.2 Catalytic reduction of methyl orange (MO)

##### 3.6.2.1 UV-vis spectral analysis and catalytic reduction behavior

The catalytic performance of biosynthesized AuNP-AI was evaluated by monitoring the catalytic reduction of MO in the presence of NaBH_4_. The catalytic reduction process was tracked using UV-Vis spectroscopy and kinetic models to assess efficiency. A direct comparison is also made between green-synthesized AuNPs and commercially available AuNPs to determine practical applicability.

**Fig. 12:**
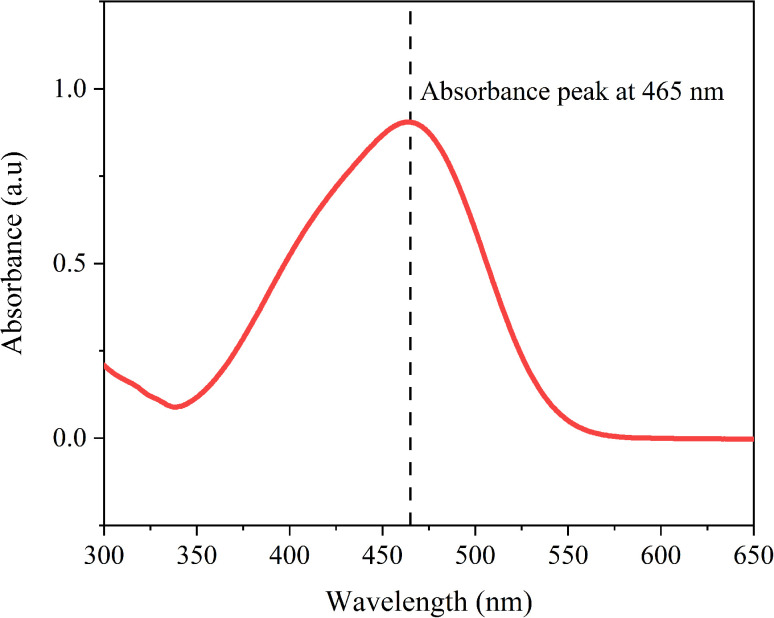
UV-Vis absorption spectrum of 15 ppm methyl orange (MO) recorded over the wavelength range of 300–650 nm. The characteristic absorption peak at 465 nm corresponds to the π–π* transition of the azo (–N=N–) chromophore, confirming the presence of MO prior to catalytic reduction.

**Fig. 13:**
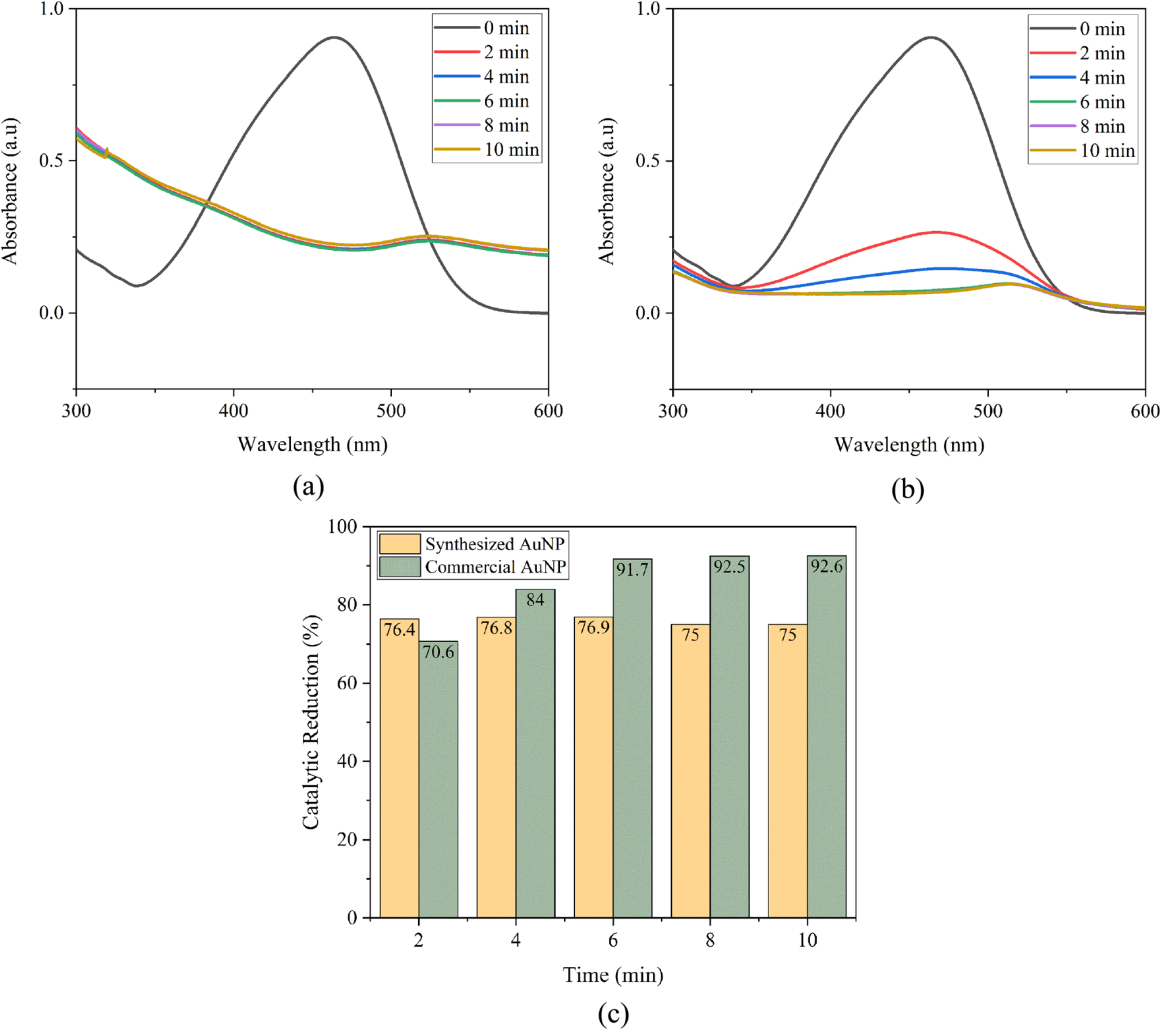
(a) Catalytic reduction of methyl orange using AuNP-AI, (b) catalytic reduction of methyl orange using commercial AuNPs, and (c) comparison of catalytic reduction efficiencies of green-synthesized and commercial AuNPs toward methyl orange.

MO shows a distinct absorbance peak around 465 nm (Fig. 12), linked to its azo (–N=N–) chromophore.^75^ When treated with NaBH_4_ alone, MO experiences minimal catalytic reduction. However, adding AuNPs, whether green-synthesized or commercial, significantly speeds up the catalytic reduction process. In the case of AuNP-AI, the UV-Vis spectra (Fig. 13a) show a rapid decrease in the 465 nm peak intensity, indicating effective catalytic reduction of MO. The peak's complete disappearance within 10 minutes demonstrates high catalytic efficiency. This effectiveness arises from gold nanoparticles' ability to transfer electrons from NaBH_4_ to MO, resulting in azo bond cleavage.^76,77^

##### 3.6.2.2 Comparative efficiency: green-synthesized *versus* commercial gold nanoparticles

The catalytic reduction of MO (Fig. 13a–c) over time was used to compare catalytic activity. Both catalysts showed significant catalytic reduction, but AuNP-AI consistently outperformed the commercial AuNPs, especially during the initial reaction period. By the 10 minute mark, AuNP-AI achieved over 75% catalytic reduction, while commercial AuNPs reached around 92% under the same conditions, calculated according to eqn (1). However, a closer inspection of the UV-Vis spectra reveals a crucial distinction: AuNP-AI led to the complete disappearance of the MO absorption peak, confirming total pollutant's catalytic reduction, whereas commercial AuNPs retained a residual shoulder despite the higher calculated catalytic reduction percentage. This discrepancy arises because the mathematical calculation of catalytic reduction efficiency accounts only for relative absorbance intensity but does not capture residual spectral features. Thus, although the calculated value is lower, the practical catalytic effectiveness of AuNP-AI is superior. This enhanced performance of AuNP-AI can be attributed to its smaller average particle size (∼11.90 nm), which provides greater surface area, the presence of bioactive phytochemicals from *Azadirachta indica* acting as capping agents that facilitate electron mediation and prevent aggregation,^78^ and more uniform and crystalline nanoparticles.^79^

##### 3.6.2.3 Kinetic analysis

**Fig. 14:**
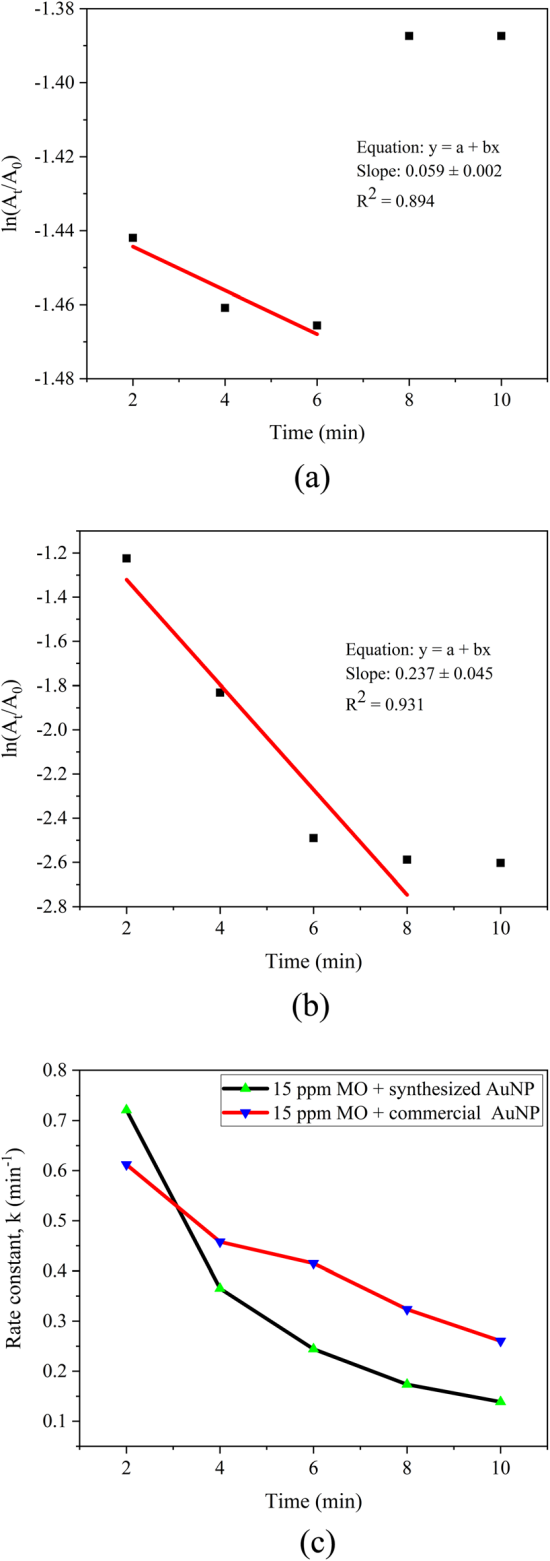
(a) Pseudo-first-order kinetic plot for methyl orange reduction using AuNP-AI, (b) pseudo-first-order kinetic plot for methyl orange reduction using commercial AuNPs, and (c) time-dependent variation of the rate constant for methyl orange reduction.

The catalytic reduction kinetics of MO were analyzed using a pseudo-first-order model, expressed as ln(*A*_*t*_/*A*_0_) *versus* time (Fig. 14a and b).^76^ The linear fitting of ln(*A*_*t*_/*A*_0_) *versus* time yielded apparent rate constants (*k*) of 0.059 ± 0.002 min^−1^ for green-synthesized AuNPs and 0.237 ± 0.045 min^−1^ for commercial AuNPs, with corresponding *R*^2^ values of 0.894 and 0.931, respectively. These values fall within the typical range reported in literature for AuNP-mediated MO reduction (0.05–0.50 min^−1^), confirming the catalytic competence of both systems.^80–82^ The slightly lower *R*^2^ values for AuNP-AI relative to commercial AuNPs reflect factors such as polydispersity, natural surface coatings, and minor aggregation, which cause non-uniform catalytic activity across particles.^83,84^

As shown in Fig. 14c, the rate constant for AuNP-AI decreased more sharply over time (from ∼0.72 min^−1^ at 2 min to ∼0.13 min^−1^ at 10 min), while commercial AuNPs maintained steadier catalytic performance (from ∼0.61 min^−1^ to ∼0.26 min^−1^). This difference reflects the high initial electron transfer efficiency of green AuNPs, followed by partial deactivation due to surface fouling or capping-related site blocking.^53,83^ To address this, more advanced kinetic models, such as the Langmuir–Hinshelwood model, could be applied in future studies to better capture the role of surface heterogeneity and adsorption-reaction dynamics.

#### 3.6.3 Catalytic reduction of 4-nitrophenol (4-NP)

##### 3.6.3.1 UV-vis spectral analysis and catalytic reduction behavior

The catalytic reduction of 4-nitrophenol to 4-aminophenol (4-AP) using NaBH_4_ was employed as a model reaction to evaluate the activity of green-synthesized AuNP-AI and commercial AuNPs. The reaction progression was tracked through UV-Vis spectroscopy and kinetic analysis.

**Fig. 15:**
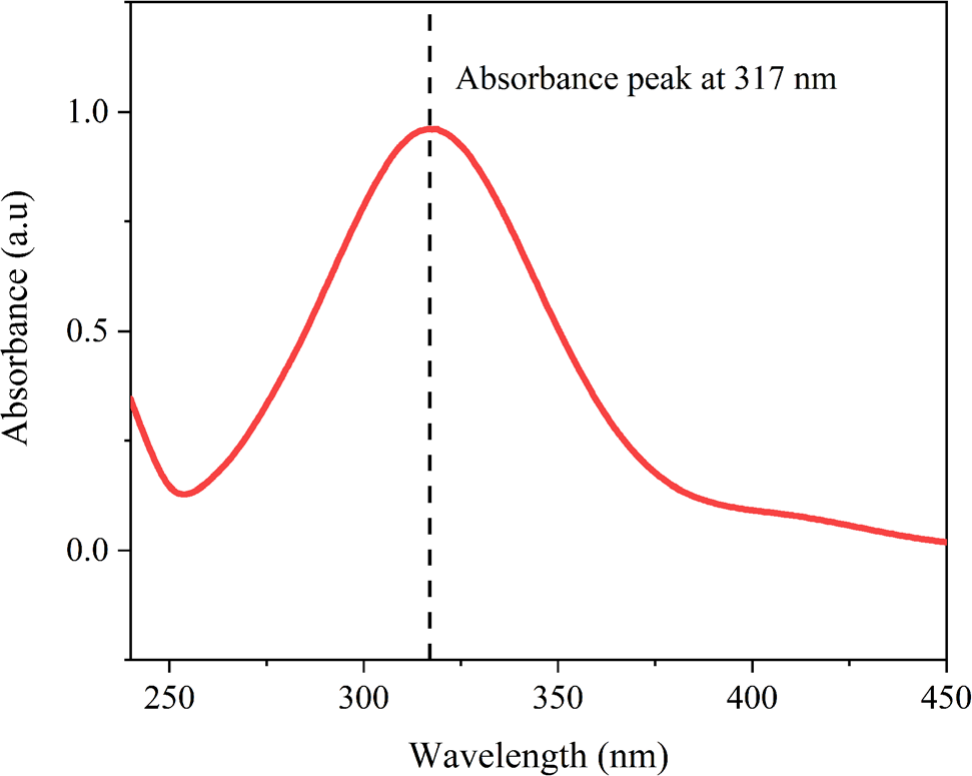
UV-Vis absorption spectrum of 4-nitrophenol (4-NP) solution recorded over the wavelength range of 200–450 nm. The characteristic absorption peak at 317 nm corresponds to the π–π* transition of neutral 4-NP in aqueous medium, serving as the reference spectrum prior to catalytic reduction.

**Fig. 16:**
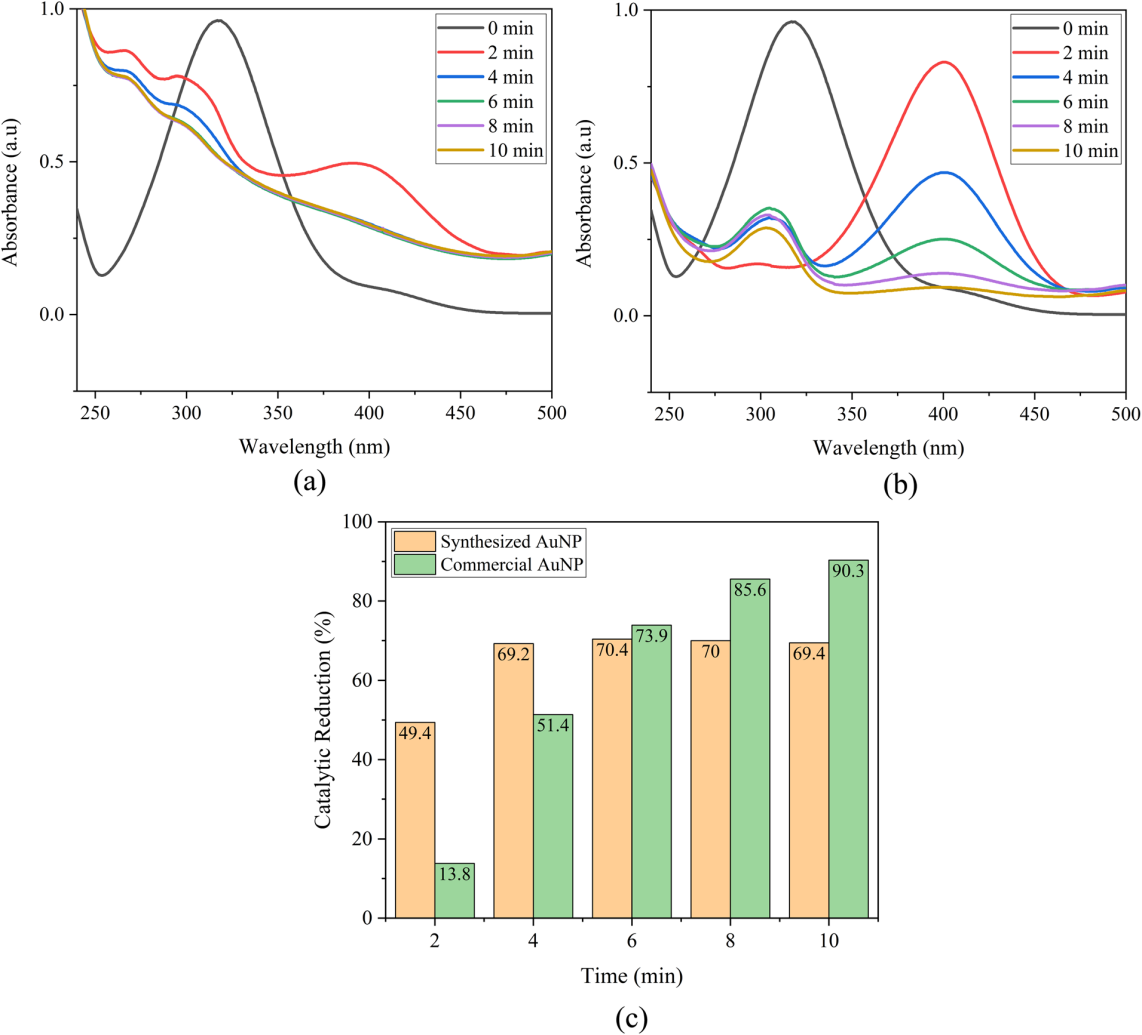
(a) Catalytic reduction of 4-nitrophenol using AuNP-AI, (b) catalytic reduction of 4-nitrophenol using commercial AuNPs, and (c) comparison of catalytic reduction efficiencies of green-synthesized and commercial AuNPs toward 4-nitrophenol.

In aqueous solution, 4-NP shows a peak at about 317 nm (Fig. 15), which shifts to 400 nm when NaBH_4_ is added due to the formation of 4-nitrophenolate ions in alkaline conditions.^85^ Without a catalyst, this peak stays the same, indicating that NaBH_4_ alone does not reduce 4-NP at a measurable rate. When AuNPs are added, a rapid decrease in the 400 nm absorbance peak occurs (Fig. 16a and b). This decrease, along with the appearance of a new peak around 300 nm, confirms successful catalytic conversion of 4-NP to 4-AP.^86^ The green-synthesized AuNP-AI effectively catalyzed this process, with nearly complete reduction in just 10 minutes, demonstrating its strong catalytic activity. This transformation serves as a benchmark test for evaluating noble metal nanocatalysts and involves surface-mediated electron transfer from BH_4_^−^ to 4-NP.^87^

##### 3.6.3.2 Comparative efficiency: green-synthesized *versus* commercial gold nanoparticles

The percent catalytic reduction of 4-NP (Fig. 16c) further illustrates the catalytic performance of the two catalysts. According to eqn (1), AuNP-AI achieved over 69% reduction of 4-NP within 10 minutes, while commercial AuNPs reached approximately 90% under the same conditions. However, a closer examination of the UV-Vis spectra highlights an important distinction: AuNP-AI led to the immediate disappearance of the characteristic absorption peak of 4-NP, confirming the complete pollutant's catalytic reduction, whereas commercial AuNPs retained a residual peak until about 8 minutes into the reaction. This again demonstrates that, despite a lower calculated catalytic reduction percentage, the practical catalytic effectiveness of AuNP-AI is superior. The enhanced activity of AuNP-AI can be attributed to its smaller particle size (∼11.90 nm), providing higher surface area, the phytochemical capping agents from *Azadirachta indica* that facilitate electron transfer and prevent aggregation, and the uniform and crystalline nature of the nanoparticles. These findings are consistent with earlier studies showing that biogenic AuNPs often display superior catalytic behavior compared to chemically synthesized counterparts due to the presence of bio-organic surface functional groups that promote pollutant adsorption and electron mediation.

##### 3.6.3.3 Kinetic analysis

**Fig. 17:**
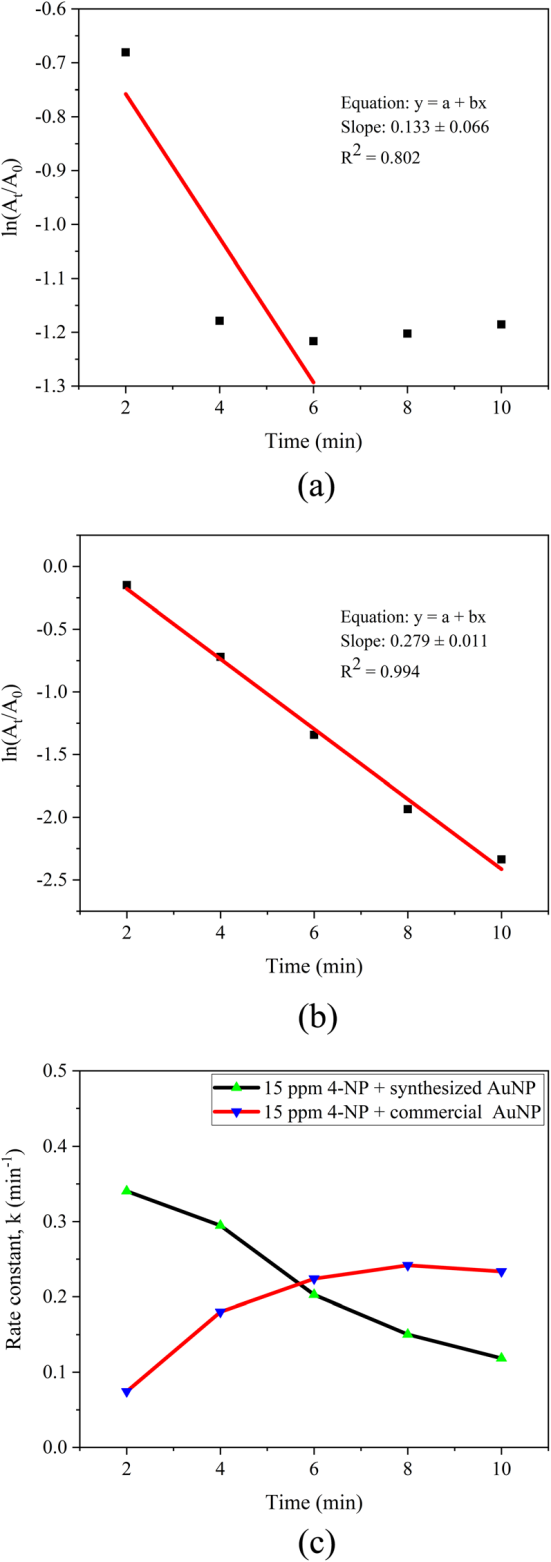
(a) Pseudo-first-order kinetic plot for 4-nitrophenol reduction using AuNP-AI, (b) pseudo-first-order kinetic plot for 4-nitrophenol reduction using commercial AuNPs, and (c) time-dependent variation of the rate constant for 4-nitrophenol reduction.

The catalytic reduction kinetics of 4-NP were also analyzed using a pseudo-first-order model, where ln(*A*_*t*_/*A*_0_) was plotted against time (Fig. 17a and b).^88^ The linear fitting of ln(*A*_*t*_/*A*_0_) *versus* time yielded apparent rate constants (*k*) of 0.133 ± 0.066 min^−1^ for green-synthesized AuNPs and 0.279 ± 0.011 min^−1^ for commercial AuNPs, with corresponding *R*^2^ values of 0.802 and 0.994, respectively. These values fall within the typical range reported in literature for AuNP-mediated 4-NP reduction (0.05–0.50 min^−1^), confirming the catalytic competence of both systems.^89,90^ The relatively lower *R*^2^ value for AuNP-AI indicates deviations from ideal kinetic behavior, which can be attributed to nanoparticle polydispersity, heterogeneous surface capping by phytochemicals, and possible aggregation, leading to non-uniform catalytic sites and variable electron transfer efficiency.^80,81,83,84^

As shown in Fig. 17c, the rate constant for AuNP-AI was higher in the early stages (∼0.34 min^−1^ at 2 min), reflecting rapid electron transfer and efficient reduction of 4-NP to 4-aminophenol. However, *k* gradually declined to ∼0.11 min^−1^ by 10 min, likely due to surface fouling by intermediates, partial active-site saturation, or nanoparticle clumping. In contrast, commercial AuNPs exhibited a slower initial rate (∼0.09 min^−1^) but maintained steadier kinetics, increasing to ∼0.24 min^−1^ by 6–8 min and stabilizing thereafter.

This crossover in catalytic dominance indicates that green AuNPs excel during the rapid initial reduction phase, while commercial AuNPs sustain more consistent activity over time. Such differences highlight the trade-off between the high reactivity but lower long-term stability of biosynthesized AuNPs and the steady, uniform performance of chemically synthesized counterparts. Future work may explore alternative kinetic models, such as Langmuir–Hinshelwood to better describe these heterogeneous surface interactions.^53,83^

#### 3.6.4 Synergistic effects

**Fig. 18:**
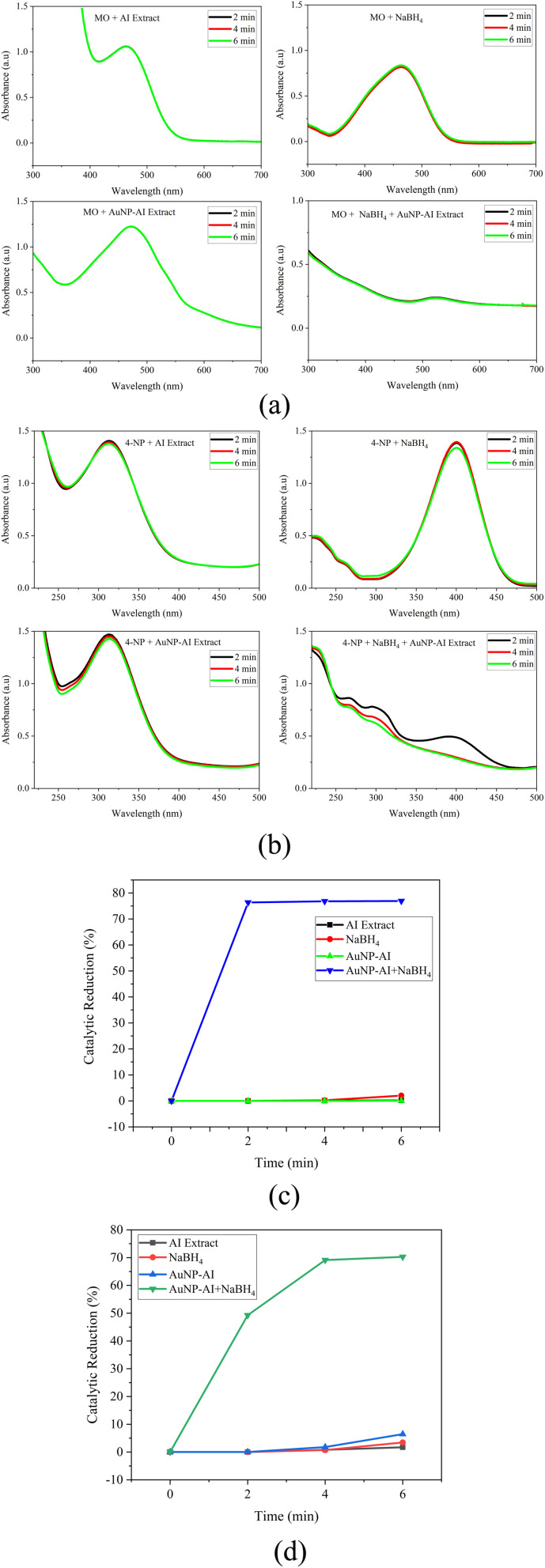
(a) Control experiments showing the effect of AI extract, NaBH_4_, and AuNP-AI on methyl orange, (b) control experiments showing the effect of AI extract, NaBH_4_, and AuNP-AI on 4-nitrophenol, (c) synergistic catalytic reduction of methyl orange by the combined AuNP-AI/NaBH_4_ system, and (d) synergistic catalytic reduction of 4-nitrophenol by the combined AuNP-AI/NaBH_4_ system.

The combined action of *Azadirachta indica* phytochemicals, green-synthesized AuNPs, and NaBH_4_ exhibited a pronounced synergistic effect in the catalytic reduction of methyl orange and 4-nitrophenol. Control experiments confirmed that NaBH_4_ alone, or NaBH_4_ in the presence of AI extract, led to negligible catalytic reduction, with almost no significant change in the absorbance spectra after 6 min (Fig. 18a and b). Similarly, neither the plant extract alone nor the AuNPs alone achieved measurable pollutant reduction, confirming that none of the individual components could effectively drive the reaction. In contrast, the catalytic system combining AuNP-AI with NaBH_4_ resulted in a rapid and complete disappearance of the absorption peak of MO, with nearly 75% catalytic reduction achieved within 6 min (Fig. 18c). A parallel trend was observed for 4-NP, where about 70% catalytic reduction occurred over the same period (Fig. 18d).

Mechanistically, NaBH_4_ acts as a hydride donor, while AuNPs serve as electron bridges, facilitating efficient electron transfer to pollutant molecules, consistent with surface-mediated reduction models, especially in 4-NP conversion to 4-aminophenol.^91^ The AI extract further contributes by capping and stabilizing the AuNPs through phytochemicals (flavonoids, terpenoids, phenolics), which introduce –OH and –COOH functional groups that enhance surface electron density and prevent nanoparticle aggregation. This improved electron shuttle is supported by findings on biogenic AuNPs and dye/nitroaromatic catalytic reduction.^92^ Additionally, the microwave-assisted synthesis produces uniform and crystalline AuNPs (∼11.90 nm), providing a larger active surface area for catalysis.^93^

This three-way synergy, NaBH_4_ as a reductant, AuNPs as an electron relay, and AI phytochemicals as stabilizers and enhancers, reduced the activation barrier and sped up the pollutant's catalytic reduction. The achieved catalytic efficiency and kinetics match reported findings on AuNP-catalyzed reductions of nitroaromatics and azo dyes.^93^

It should be noted that replicate control experiments were performed primarily for the green-synthesized AuNPs, while identical replicate testing for commercial AuNPs was limited due to material and resource constraints. Although all catalytic comparisons were conducted under identical reaction conditions, future studies will include full replicate analyses for commercial catalysts to further strengthen statistical robustness and comparative assessment.

#### 3.6.5 Reusability test of AuNP-AI nanocatalyst

**Fig. 19:**
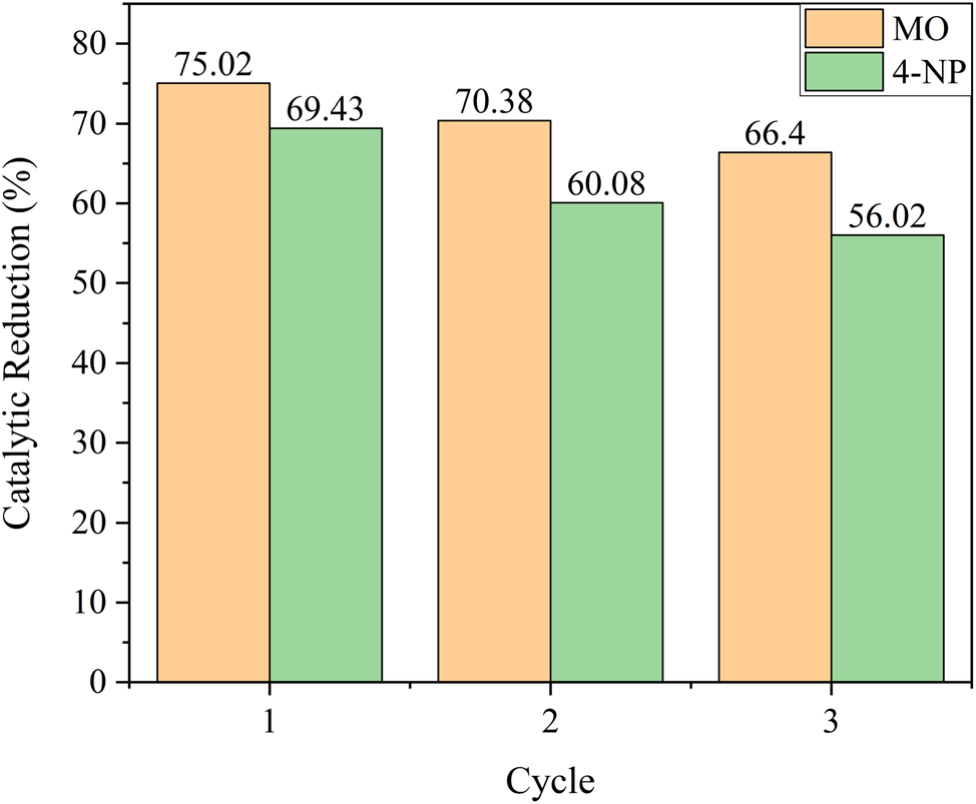
Reusability performance of AuNP-AI nanocatalyst over successive catalytic reduction cycles for methyl orange and 4-nitrophenol.

The recyclability of the green-synthesized AuNP-AI catalyst was evaluated over three consecutive cycles using MO and 4-NP as pollutants. As shown in Fig. 19, the catalytic reduction efficiency for MO decreased slightly from 75.02% in the first cycle to 70.38% and 66.40% in the second and third cycles, corresponding to retention rates of 100%, 93.8%, and 88.5%, respectively. A similar trend was observed for 4-NP (Fig. 19), with catalytic reduction efficiencies of 69.43%, 60.08%, and 56.02% in the first, second, and third cycles, giving retention rates of 100%, 86.5%, and 80.6%, respectively. The gradual decline in performance can be attributed to partial loss of catalyst during recovery steps, possible surface fouling by reaction intermediates, and minor aggregation of nanoparticles, as reported in similar green synthesis studies.^94^ Nevertheless, the catalyst maintained more than 80% of its initial activity after three cycles, demonstrating appreciable stability and reusability. These results underscore the potential of microwave-assisted green-synthesized AuNPs as sustainable nanocatalysts for wastewater treatment, while highlighting the need for future optimization of recovery methods, long-term recyclability testing, and detailed mechanistic analysis of catalyst deactivation pathways.

#### 3.6.6 Stability test of AuNP-AI nanocatalyst

**Fig. 20:**
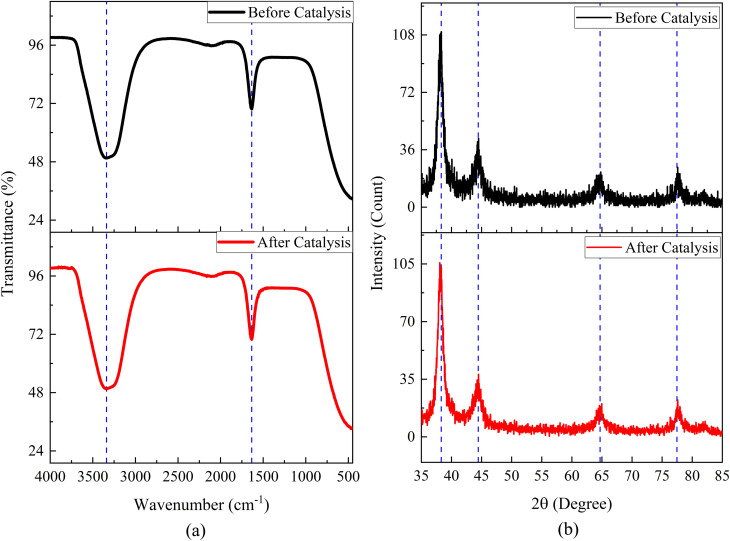
(a) FTIR spectra of AuNP-AI before and after catalytic reduction, and (b) XRD patterns of AuNP-AI before and after catalytic reduction, demonstrating structural and chemical stability.

To assess the structural and chemical stability of the biosynthesized AuNP-AI catalyst, we compared FTIR and XRD spectra collected before and after catalytic reduction cycles. The FTIR spectra (Fig. 20a) displayed essentially identical peak positions corresponding to functional groups (*e.g.*, –OH, C=O), indicating that the primary phytochemical ligands remained bound to the nanoparticle surface and no new chemical species or catalytic reduction by-products emerged. Similarly, in Fig. 20b, the XRD patterns after catalysis preserved the characteristic face-centered cubic gold diffraction peaks, with only a slight decrease in intensity, likely due to minor loss of crystallinity or partial diminution of scattering volume. The absence of new diffraction peaks rules out the formation of Au oxides or other crystalline contaminants. Together, these results provide strong evidence that the AuNP-AI catalyst maintains its structural integrity, retains its ligand shell, and does not undergo significant chemical transformation during the catalytic cycles. This high stability parallels observations from green-synthesized AuNPs in the literature, where minimal structural change is regarded as a hallmark of robustness in nanocatalysts.^95^

#### 3.6.7 Catalytic mechanism of methyl orange and 4-nitrophenol by AuNP-AI extract

The catalytic reduction of methyl orange and 4-nitrophenol using eco-friendly synthesized AuNPs is generally understood to proceed *via* a surface-mediated electron relay process, in which gold nanoparticles are proposed to act as electron shuttles between the reducing agent (NaBH_4_) and the target organic pollutants. Sodium borohydride alone can thermodynamically reduce MO and 4-NP but does so slowly; therefore, the presence of AuNPs is widely reported to accelerate the reaction by lowering the apparent activation barrier and facilitating electron transfer from BH_4_^−^ to the dye molecules.^96,97^

In the present study, AuNPs synthesized using *Azadirachta indica* extract are expected to provide both reduction and stabilization functions. Phytochemicals such as flavonoids and terpenoids present in the AI extract are known from literature to participate in the reduction of Au^3+^ to Au^0^ and are suggested to contribute to nanoparticle stabilization, resulting in high surface area and catalytically accessible sites that are favorable for reduction reactions.^98^

For methyl orange, the reduction pathway is commonly described as involving electron-induced cleavage of the azo (–N=N–) bond, leading to decolorization and the formation of aromatic amine derivatives.^7^ In the case of 4-nitrophenol, the reaction is conventionally monitored by a bathochromic shift of the absorption peak from ∼317 nm to ∼400 nm upon addition of NaBH_4_, corresponding to the formation of 4-nitrophenolate ions. In the presence of AuNPs, this species is reported to undergo stepwise hydrogenation to yield 4-aminophenol.^99–101^

In this work, the catalytic reduction of 4-NP to 4-aminophenol and the reduction of MO to colorless products were significantly accelerated in the presence of NaBH_4_ and AuNP-AI compared to control systems. The green-synthesized AuNPs are proposed to function as effective catalysts by mediating electron transfer from BH_4_^−^ to the pollutant molecules, consistent with established models of noble-metal-assisted reduction reactions.

Based on widely accepted literature mechanisms, the following reaction steps are proposed:

For MO:

i MO + BH_4_^−^ → activated MO^−^

ii BH_4_^−^ + AuNPs → AuNPs (e^−^) + H_2_↑

iii MO^−^ + e^−^ → aromatic amine derivatives

iv Overall: MO + NaBH_4_ + AuNPs → reduced amine products

For 4-NP:

i 4-NP + OH^−^ → 4-nitrophenolate

ii BH_4_^−^ + AuNPs → AuNPs (e^−^) + H_2_↑

iii 4-nitrophenolate + e^−^ → 4-hydroxylaminophenol

iv 4-hydroxylaminophenol + e^−^ → 4-aminophenol

v Overall: 4-NP + NaBH_4_ + AuNPs → 4-aminophenol

These sequential reduction pathways are presented as proposed mechanisms derived from established AuNP-mediated catalytic reduction models and are consistent with previous reports on NaBH_4_-assisted reduction of azo dyes and nitroaromatic compounds.^88,102,103^

It is important to note that, due to resource limitations, reaction intermediates were not directly identified in this study (*e.g.*, by HPLC, LC-MS, XPS, or *in situ* FTIR). Accordingly, the mechanistic pathways discussed above should be interpreted as plausible and literature-supported, rather than experimentally confirmed within the present work.^89,90,104,105^ Future studies incorporating surface-specific and intermediate-resolved analytical techniques are recommended to experimentally validate these mechanistic aspects.

#### 3.6.8 Selection of 15 ppm pollutant concentration for catalytic studies

To identify the optimal substrate concentration that best reflects the catalytic capabilities of the synthesized AuNP-AI, comparative catalytic reduction experiments were conducted using 10 ppm and 15 ppm solutions of both MO and 4-NP. The results showed that while both concentrations underwent catalytic reduction, the 15 ppm solutions consistently demonstrated a higher percentage of catalytic reduction within the same reaction period when treated with AuNP-AI and NaBH_4_ (Table 4). For example, the MO solution at 15 ppm achieved higher catalytic reduction rates and clearer endpoints compared to the 10 ppm solution, which risked plateauing within the noise threshold, consistent with observations reported across similar studies of dye catalytic reduction kinetics.^106^ Similarly, in the case of 4-NP, the 15 ppm solution exhibited improved reaction dynamics and more definitive pseudo-first-order decay curves, allowing for more reliable kinetic fitting. Therefore, 15 ppm was chosen as the working concentration for all catalytic and mechanistic analyses, as it provided both strong signal changes and clear differentiation of catalytic performance under typical pollutant loading conditions.

**Table 4:** Catalytic reduction efficiency of methyl orange and 4-nitrophenol at different initial pollutant concentrations using AuNP-AI

Pollutants	Concentration	Catalytic reduction (%)		Correlation coefficient (*R*^2^)	
		Green AuNPs	Commercial AuNPs	Green AuNPs	Commercial AuNPs
MO	10 ppm	64.17	90.40	0.738	0.939
	15 ppm	75.02	92.59	0.894	0.931
4-NP	10 ppm	65.85	88.44	0.891	0.983
	15 ppm	69.43	90.33	0.802	0.994

## 4 Conclusions

This study demonstrates a fast, sustainable, and eco-friendly method for synthesizing gold nanoparticles *via* microwave-assisted green synthesis using *Azadirachta indica* leaf extract as both reducing and stabilizing agent. Optimization of synthesis parameters produced uniformly dispersed, crystalline AuNPs with an average particle size of 11.90 ± 2.84 nm and excellent structural and optical stability. Catalytic evaluation showed that the green-synthesized AuNPs effectively reduced methyl orange and 4-nitrophenol through a synergistic electron relay mechanism, in which AuNPs, NaBH_4_, and phytochemical capping agents collectively accelerated pollutant's reduction. A key novelty of this work lies in the direct comparative study of biogenic and commercial AuNPs, which revealed that bio-derived AuNPs performed as well as, and in some cases better than, their commercial counterparts.

Despite these promising findings, several limitations remain. The minimum effective catalyst concentration and the yield of green synthesis relative to commercial production were not determined in this study. Detailed mechanistic investigations using advanced techniques such as HPLC, XPS, or LC-MS could not be conducted due to resource constraints. Likewise, systematic control experiments (precursor without extract, extract without microwave, microwave without extract) to fully decouple the contributions of microwave irradiation, phytochemicals, and NaBH_4_ were not included, and only a single catalyst dose was tested, without exploring dosage-dependent effects. These aspects will be systematically addressed in future work.

Overall, by combining green chemistry principles with microwave technology, this study establishes a rapid, energy-efficient, and scalable strategy to produce high-performance nanocatalysts. The comparative and mechanistic insights presented here highlight the practical potential of green AuNPs for wastewater treatment, while also providing a foundation for future studies aimed at refining synthesis efficiency, expanding mechanistic understanding, and validating long-term catalytic applicability.

## Author contributions

Md. Abdus Sabur: writing – review and editing, writing – original draft, funding acquisition, methodology, investigation, formal analysis, data curation, conceptualization, software, supervision. Ishraque Karim: writing – original draft, methodology, formal analysis, data curation, software. Aninda Nafis Ahmed: review, resource, and project administration.

## Conflicts of interest

The authors confirm that they have no financial or personal conflicts of interest that could have affected the research in this study.

## Data Availability

Although sufficient data are presented in tables and figures, all authors state that additional data will be provided upon request.
